# Brightness-equalized quantum dots

**DOI:** 10.1038/ncomms9210

**Published:** 2015-10-05

**Authors:** Sung Jun Lim, Mohammad U. Zahid, Phuong Le, Liang Ma, David Entenberg, Allison S. Harney, John Condeelis, Andrew M. Smith

**Affiliations:** 1 Department of Bioengineering, University of Illinois at Urbana-Champaign, Urbana, Illinois 61801, USA; 2 Micro and Nanotechnology Laboratory, University of Illinois at Urbana-Champaign, Urbana, Illinois 61801, USA; 3 Department of Materials Science and Engineering, University of Illinois at Urbana-Champaign, Urbana, Illinois 61801, USA; 4 Department of Anatomy and Structural Biology, Albert Einstein College of Medicine of Yeshiva University, Bronx, New York 10461, USA; 5 Integrated Imaging Program, Albert Einstein College of Medicine of Yeshiva University, Bronx, New York 10461, USA; 6 Gruss-Lipper Biophotonics Center, Albert Einstein College of Medicine of Yeshiva University, Bronx, New York 10461, USA; 7 Department of Radiology, Albert Einstein College of Medicine of Yeshiva University, Bronx, New York 10461, USA

## Abstract

As molecular labels for cells and tissues, fluorescent probes have shaped our understanding of biological structures and processes. However, their capacity for quantitative analysis is limited because photon emission rates from multicolour fluorophores are dissimilar, unstable and often unpredictable, which obscures correlations between measured fluorescence and molecular concentration. Here we introduce a new class of light-emitting quantum dots with tunable and equalized fluorescence brightness across a broad range of colours. The key feature is independent tunability of emission wavelength, extinction coefficient and quantum yield through distinct structural domains in the nanocrystal. Precise tuning eliminates a 100-fold red-to-green brightness mismatch of size-tuned quantum dots at the ensemble and single-particle levels, which substantially improves quantitative imaging accuracy in biological tissue. We anticipate that these materials engineering principles will vastly expand the optical engineering landscape of fluorescent probes, facilitate quantitative multicolour imaging in living tissue and improve colour tuning in light-emitting devices.

## Abstract (web summary)

Quantum dots with different size emit light at different wavelengths but also different brightness, which complicates analysis of fluorescence images. Here, the authors synthesize multicolour brightness-equalized quantum dots by controlling the composition and structure of core-shell HgCdSeS-CdZnS nanocrystals.

## Graphical abstract

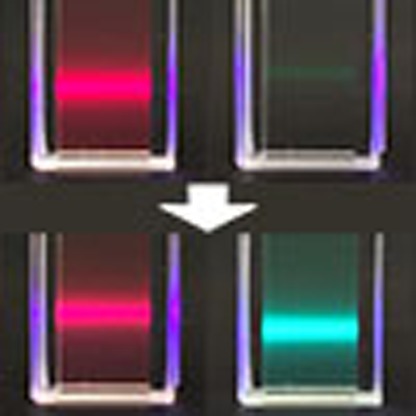

Semiconductor quantum dots (QDs) are the subject of a diverse range of fundamental and applied research efforts in biomedical imaging, light-emitting devices, solar cells and quantum computing^1^,^2^,^3^,^4^,^5^,^6^,^7^,^8^,^9^,^10^,^11^,^12^. These light-absorbing, light-emitting nanocrystals provide numerous optical and electronic properties that are not available from other materials. In particular for molecular and cellular imaging applications, QDs have a unique combination of bright and stable fluorescent light emission, widely tunable and pure emission colours and broadband excitation. In recent years, these properties have provided a means to image and track proteins and nucleic acids at the single-molecule level for long durations and to multiplex the detection of a large number of molecules and biomolecular processes simultaneously without crosstalk^13^,^14^,^15^. The critical capacity to tune the emission colour of a QD derives from the quantum confinement effect, whereby the nanocrystal dimensions (size and shape) dictate the energies of excited-state charge carriers (electrons and holes)^16^,^17^,^18^,^19^. Reducing the nanocrystal size confines the charge carriers to a smaller region in space, which increases their energies, widens the electronic bandgap and shifts the absorption and emission spectra to higher energy (shorter wavelength). Through synthetic advances over the last two decades, size-tunable QDs can now be readily prepared from a variety of materials, which has yielded emitters throughout the near-ultraviolet, visible, near-infrared and mid-infrared spectra with fluorescence quantum yields (*QY*) approaching 100% (refs 20, 21, 22, 23, 24, 25).

An undesirable consequence of exploiting the quantum confinement effect for spectral tuning is that different colours of emitters are necessarily dissimilar in fluorescence brightness. This is primarily due to differences in extinction coefficients (*ε*): the size determines the number of constituent atoms and bonds, which are the fundamental units of collective electronic oscillation mediating light absorption and extinction. More atoms per particle provide more bonding electrons, which leads to higher oscillator strengths and higher light collecting efficiency per particle^26^,^27^. Thus, for a spherical nanocrystal with radius *r*, *ε* scales approximately with volume (*ε* ∝ *r*^3^) in single-photon excitation mode. Light absorption results in an excited state electron that then decays to its ground states by converting its energy to fluorescent light; the efficiency of this process is the *QY*. Thus, the relative fluorescence brightness, *B*_rel_, is simply the product^28^:




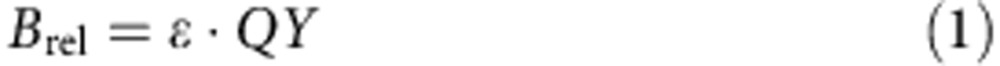




If *QY* is similar for each QD colour, the brightness can differ by orders of magnitude across a small spectral range simply because the extinction coefficient is intrinsically coupled to the size and thus the emission wavelength (*λ*_em_). This effect is demonstrated in the top panels of Fig. 1. Prototypical size-tuned QDs (ST-QDs; depicted in Fig. 1a) are composed of CdSe cores with a size that largely determines *λ*_em_ and *ε*. The core is coated with a shell composed of CdS, ZnS or their alloys, which provides electronic insulation and almost entirely dictates *QY*, but can also contribute significantly to *λ*_em_ and *ε*. As shown in Fig. 1b, four sizes of ST-QDs thus have very dissimilar extinction coefficients that increase with size; therefore, upon excitation by short-wavelength light (for example, 400 nm), redder QDs have greater brightness compared with bluer QDs. As shown in Fig. 1c, QDs emitting at 650 nm have a ∼48-fold greater fluorescence brightness compared with 520 nm QDs based on extinction coefficient alone^29^. Dissimilarities in *QY* tend to further exacerbate this effect (*vide infra*). In two-photon excitation mode, the mismatch in relative brightness is even wider, on the order of 100- to 200-fold from green to red, as *ε* scales proportionally with *r*^4^ (ref. 30). Two-photon excitation is critically important for bioimaging because long-wavelength illumination intrinsic to the modality both enhances depth penetration through thick tissue and reduces tissue damage^31^,^32^,^33^. The colour-dependent optical mismatch of QDs is even more pronounced in biological media, as small QDs with the lowest fluorescence intensity overlap the most with blue/green autofluorescence of biological molecules, reducing their detection threshold as they are buried in a noisy background and shoulders of spectrally adjacent red emitters that are much brighter^34^,^35^. The consequence of this mismatch is that the multiplexing advantages of QDs are substantially diminished when detecting, imaging and tracking biomolecular analytes, and only a limited optical spectrum is regularly utilized.

Here we describe the development of brightness-equalized QDs (BE-QDs) with matched fluorescence brightness across a broad spectrum of colours in the visible and near-infrared. The general concept is to decouple the three key optical parameters, *λ*_em_, *ε* and *QY*, to the greatest extent possible by allowing them to derive from independent structural domains in the nanocrystal. Figure 1d depicts the 3-domain core/shell/shell BE-QD structure. We replace conventional CdSe binary cores that have a fixed bulk bandgap (1.76 eV) with ternary CdSe_*y*_S_1-*y*_ or Hg_*x*_Cd_1-*x*_Se alloys or quaternary Hg_*x*_Cd_1-*x*_Se_*y*_S_1-*y*_ alloys that have continuously tunable bulk bandgaps from 0 to 2.5 eV. This allows us to adjust the bandgap, and thus emission wavelength, without changing the nanocrystal size. Because these size-matched cores have a similar number of atoms, the extinction coefficients are intrinsically similar and can be matched precisely across a broad range of excitation spectra by epitaxial growth of a strongly absorbing shell material (CdS) using efficient deposition processes. In a final step, the overgrowth of a wide-bandgap ZnS shell normalizes the *QY* values even after transfer to oxidizing conditions in aqueous solution, with little impact on extinction. As a result, *λ*_em_, *ε* and *QY* are decoupled and can be independently adjusted to vastly expand the optical properties of QD emitters, yielding a degree of parametric tunability that is currently not available from any other type of material. We demonstrate that this leads to normalization of brightness for QDs emitting across a wide wavelength range from 500 to 800 nm with excitation between 350 and 450 nm. Brightness equalization is observed at both the ensemble level and the single-molecule level as well as under two-photon excitation conditions, which we show translates to improved quantitative imaging capabilities in complex biological tissue.

## Results

### Matching extinction coefficients

To match *ε* between different QD colours, we first measured the wavelength-dependent *ε* values for individual materials used in the different structural domains of ST-QD and BE-QD. Figure 2 depicts *ε*-spectra for three nanocrystalline materials: size-tunable core materials (CdSe), composition-tunable core materials (Hg_*x*_Cd_1-*x*_Se_*y*_S_1-*y*_) and shell material used for extinction matching (CdS). CdS, CdSe and CdSe_*y*_S_1-*y*_ were prepared by reproducible non-injection heat-up methods and Hg_*x*_Cd_1-*x*_Se(S) was synthesized from a pre-formed CdSe(S) core with Hg added through cation exchange in a controllable second step. Values of *ε* were measured through a combination of absorption spectrophotometry, transmission electron microscopy (TEM) and inductively coupled plasma optical emission spectrometry (ICP-OES), as described in the Methods section. Figure 2a shows *ε*-spectra for three sizes of binary CdSe nanocrystals; absorption spectra redshift with increasing diameter, asymptotically approaching the bulk CdSe bandgap (*E*_g_=1.76 eV or 730 nm). Emission spectra (not shown) are close in wavelength to the longest wavelength peak in these spectra. The depicted ∼100-nm redshift is accompanied by an approximately five- to sixfold increase in *ε* at short wavelengths (300–400 nm). Figure 2b depicts the result when the size is fixed and the wavelength is tuned by composition. Two important effects are clear: the tunable range is very wide (here 400–800 nm) and the extinction coefficients between different colours are closer (one- to twofold, depending on the specific colours considered). Figure 2c shows *ε*-spectra for nanocrystals composed of the binary shell material CdS, yielding similar size-tunable attributes as the CdSe core but with a substantial blueshift because of the larger bulk CdS bandgap (*E*_g_=2.5 eV or 520 nm). Importantly, although there is a large dissimilarity in *ε* between different CdS and CdSe sizes, the absorption coefficient, *α* (shown in the insets), is fairly constant with size. The parameter *α* provides a measure of the light-absorption capacity per atom or equivalently per unit cell in the crystal:




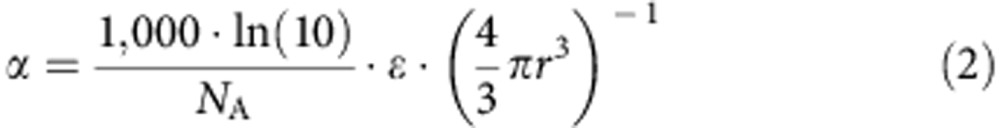




The independence of absorption cross-section at high energy (300–400 nm) for QDs is a well-known attribute of quantum confinement, which mostly affects band-edge energy levels^26^,^36^,^37^. These *α* data provided in the inset thus provide a metric for the quantity of extinction per atom of Cd/S deposited as shell material, and predict a linear increase in extinction coefficient in the low-wavelength spectral range with increasing shell volume.

We are most interested in equalizing QD extinction coefficients in the 350–450 nm spectral range, a window allowing excitation of a broad range of colours in the visible and near-infrared, and corresponding to the 700–900 nm two-photon band commonly employed with standard femtosecond laser systems. To do so, we epitaxially deposited CdS shells on pre-formed cores, with the expectations that (i) the CdS domain will provide a predictable and continuously tunable quantity of *ε* per particle and (ii) the wide bandgap CdS shell will increase *QY* through electronic insulation. First we discuss the impact on *ε*. Shells were grown homogeneously, layer-by-layer, in increments of 0.8 lattice monolayers (MLs), using highly reactive cadmium oleate and bis(trimethylsilyl)sulfide precursors to ensure quantitative deposition without introducing new CdS nuclei, as confirmed by TEM (see Supplementary Fig. 1 for details). Figure 3 shows the effects on *ε*, comparing the results when either using two colours of size-tuned cores (left columns: CdSe diameters of 2.0 or 4.3 nm) or when using two colours of composition-tuned cores (right columns: Hg_*x*_Cd_1-*x*_Se_*y*_S_1-*y*_) with the same size. For all core materials, CdS deposition substantially increased *ε* at wavelengths shorter than 500 nm (see Fig. 3a–d for details). Figure 3e,g summarizes the trends for *ε* at 400 nm, highlighting extinction isolines (dotted lines) that connect different QD colours with specific shell thicknesses at which the two *ε* values match. Whereas the QDs generated from size-tuned CdSe cores require very different quantities of shell deposition for matching different QD colours, ternary alloy cores initially have similar extinction coefficients, so the extinction matching process is greatly simplified, as extinction values at 400 nm nearly matched throughout the CdS shell growth process. As depicted in Fig. 3f,h, pairs of QDs extinction-matched at 400 nm are also matched over a broad range of spectral wavelengths between about 300 and 450 nm, allowing equivalent excitation efficiency using a wide range of excitation sources. In the insets, spectra of extinction-matched QD pairs are divided to emphasize the uniformity of *ε* over a wide range of wavelengths; this uniformity increases with growth of thicker shells as the CdS domain dominates the spectra, washing out differences arising from dissimilar cores. Note that for conventional CdSe/CdZnS ST-QDs, similar plots show differences on the order of 40-fold for different colours (see Supplementary Fig. 2 for details).

Figure 3i,j depicts the most important differences between the use of size- and composition-tuned cores for extinction matching: size-tuned cores provide little capacity for emission wavelength tuning compared with those based on alloyed cores. The maximum wavelength separation we could achieve for extinction-matched QDs using size-tuned cores was 35 nm because of a larger redshift induced by the CdS shell growth on smaller cores, arising from electron tunnelling into the shell^38^. Thus, the ∼5 ML required to equalize the small core *ε* to that of the red core decreased the wavelength difference drastically. However, we were able to achieve >150 nm spectral tunability for the ternary alloy cores, which can be further expanded into the near-infrared and into the blue spectra. This is because the extinction coefficients are similar initially, so shell growth induces similar redshifts for all of the samples. In addition, this allows the preparation of extinction-matched QDs that are more compact, a widely desirable attribute for many bioimaging applications^39^,^40^,^41^,^42^,^43^. In principle, it should be possible to achieve wider spectral tunability with binary cores by employing a shell material for which electron confinement in the core is enhanced, which would reduce spectral shifting with shell growth. However, the best materials that satisfy this requirement for II–VI cores (for example, ZnSe or ZnS) have larger bandgaps and smaller lattice constants than CdS, which yield a shorter onset wavelength for extinction enhancement (<450 nm) and lower *QY* because of lattice mismatch-induced defect formation. With the use of an alloyed or gradient shell material, it may be possible to balance all of these effects, but the structures may be more difficult to characterize and generate consistently.

### Matching *QY* and brightness

The *QY* of a semiconductor QD is largely a function of surface traps, which are localized electrostatic charges arising from non-passivated bonding orbitals that provide non-radiative decay pathways that quench fluorescence emission^18^,^44^,^45^. Although still poorly understood, it is well-known that the overgrowth of a larger bandgap shell increases *QY* by serving as an electronic insulator to reduce electron and hole wavefunction overlap with surface traps. Thus, CdS is a commonly used shell material for CdSe core nanocrystals as it strongly confines the hole to prevent access to anionic trap sites on the surface, enabling the generation of QDs with near unity *QY* at room temperature in a variety of solvents^43^,^46^,^47^. However, it is not sufficient alone unless thick shells are deposited, as much of the *QY* boost is lost after dispersion in aqueous solution because of the introduction of new surface traps from possible oxidizing adsorbates (see Supplementary Fig. 3 for details). Figure 4a,b depicts trends in *QY* during CdS shell growth in organic solvents; the *x* axes of these plots are the extinction coefficient values derived from Fig. 3. On these plots, *QY* increases initially with shell growth, and intersecting points can be found between any two QD pairs, where they are ‘brightness matched,’ as *ε* and *QY* values are each equal. These trends provide a simple method to achieve brightness-matched QDs in organic solvents, however, these results do not translate after transfer to aqueous solution unless thick shells are grown, and bluer QDs exhibited non-monotonic increases in *QY* during shell growth. These problems can be overcome by deposition of a second concentric shell of ZnS, which has an even wider bandgap (3.7 eV) and strongly confines the electron, but only has a small impact on the extinction coefficient between 300 and 500 nm (<10% change). Figure 4c,d depicts the change in QD brightness with ZnS deposition: QDs based on size-tuned CdSe cores are brightness equalized after deposition of two or three MLs of ZnS, and the QDs based on composition-tuned core are brightness equalized after different quantities of shell deposition. Cores with wider bandgaps required thicker shells, likely due to a lower degree of insulation provided by the shell material. Figure 4e,f depicts ‘brightness spectra’ of *QY* × *ε* versus excitation wavelength for different QD colours after capping with ZnS, demonstrating brightness equalization over the 350- to 450-nm excitation range and spectrally uniform *QY* when exciting below 480 nm (see Supplementary Figs 4 and 5 for details). Similar plots for conventional ST-QDs show a mismatch in brightness in the range of 93-fold, depending on the wavelength of excitation (see Supplementary Fig. 6 for details). BE-QDs could be generated based on size-tunable CdSe cores or alloys, but again, alloys provide a much wider range of spectral tunability.

### Synthesis reproducibility

To ensure broad utility of this methodology to expand the optical parameters of quantum-confined colloidal particles, it is critical that the individual processes involved are highly reproducible. We explored the reproducibility by performing three independent replicates of each step involved in the generation of sets of BE-QDs with three different emission wavelengths. We note that our chemical processes were chosen specifically for high reproducibility, employing a heat-up core synthesis process rather than an injection-based nucleation method^48^, and using shell reagents that are highly reactive and pure such that the observed shell deposition was efficient and matched expected values^49^. Supplementary Figs 7–9 display the data collected from these experiments. Hg_*x*_Cd_1-*x*_Se_*y*_S_1-*y*_ cores with three different compositions were synthesized independently three times using the two-step heat-up and mercury alloying process. These syntheses were highly reproducible: the standard deviation (s.d.) in wavelength for the first exciton absorption peak was between 0.58 and 1.2 nm for each colour and the relative standard deviation (RSD) for generating a specific *ε* value at 400 nm was between 0.3 and 2.3% for each colour. These *ε* values between different colour batches were significantly different, with a *P*-value of 1.6 × 10^−7^ (one-way analysis of variation). The extinction coefficients were then equalized across the three colours through CdS shell growth (3.2 ML), yielding 1.2–2.8% RSD within colour batches and a *P*-value of 15% between different colour batches for the process. The *QY* values were then equalized through ZnS shell growth (2.4 ML), with values in chloroform varying with a 2.6–3.1% RSD within batches and *P*-value of 78% between different colour batches. When these particles were transferred to water, the *QY* differences widened in dispersion, with 4.3–6.7% RSD within batches and *P*-value of 73% between different colour batches. The resulting QDs in aqueous solution had 5.5–7.7% RSD in brightness values and *P*-value of 39% between different colour batches, with a maximum of 3.0 nm s.d. in emission wavelength. The most variable individual process for a specific colour was *QY* equalization and the most variable process for brightness equalization between different colours was *ε* equalization. Overall, we consider this to be a very high level of reproducibility and can be further improved by continuously monitoring changes in *ε* and *QY* during the growth process.

We also found that these nanocrystals undergo an ‘aging’ process as an ensemble by slightly changing in brightness over time in aqueous solution, an effect previously observed for other QDs^50^. As shown in Supplementary Fig. 10, after 8 months in storage the brightness values of green and red BE-QDs decreased by 30–40%, yielding relative brightness values for the pair that changed from 1.0-fold (equalized) to 1.17-fold. In comparison, ST-QDs with the same wavelengths as the BE-QDs diverged in brightness to a much greater degree over after 8 months in storage, with the green QDs decreasing in brightness by 59% and the red QDs increasing by 14%. The resulting brightness values were initially mismatched by 93-fold and increased in difference to 260-fold after 8 months. We attribute the improved similarity in stability of the BE-QDs to the use of shells with nearly identical composition and thickness, which are critical contributors to both *QY* and extinction.

We further investigated how ligand coating chemistry impacts the brightness of BE-QDs (see Supplementary Fig. 11 for details). For the majority of this work, we employed amphiphilic polymers to coat the nanocrystals in water, which allow the retention of the original ligands from shell synthesis on the nanocrystal surface. However, recently multidentate polymers and thin ligand coatings have been employed to prepare QDs with a smaller hydrodynamic size, which is more useful for many biomolecular detection applications^39^,^40^,^41^,^42^,^43^,^51^,^52^,^53^. With multidentate polymers, the *QY* values were slightly lower and the resulting brightness values were slightly more variable (9.4% RSD in average brightness between three colours). With thiol-based ligands, *QYs* were much lower and the brightness differences between colours were more pronounced (68% average RSD between colours), as green BE-QDs exhibited drastically reduced *QY* (20-fold reduction) compared with red ones (3-fold reduction). Further development of the shell material to prevent leakage of charge carriers to the surface for all of the colours evenly may eliminate this effect for thiol-based ligands.

### Single-molecule brightness

We compared the brightness of two colours of BE-QDs (525 and 650 nm) and two colours of conventional CdSe/CdZnS ST-QDs (525 and 655 nm) at the ensemble and single-molecule levels. TEM showed that all QDs were relatively homogeneous in diameter (see Supplementary Fig. 12 for details). All QDs were phase transferred to phosphate-buffered saline (PBS) using the same polymeric surface coating before measurement. Figure 5 depicts the ensemble fluorescence spectra of these QDs at identical molar concentrations with excitation at 400 nm, in addition to their spectrally integrated fluorescence intensities. For the ST-QDs, the red QDs were 93-fold brighter than the green QDs. For the BE-QDs, the brightness values were nearly identical. We spin-coated dilute suspensions of these QDs onto glass coverslips and examined their brightness at the single-molecule level using epifluorescence microscopy with 400 nm excitation. Examples of integrated movie frames are shown in the right of Fig. 5. QDs were imaged at 19.4 frames per second and all QDs exhibited fluorescence intermittency (blinking). To determine the brightness of the ‘on’ fluorescence state only and to eliminate particle aggregates from consideration, histograms of brightness for each QD were fit to a sum a noise peak (a Gaussian) and a QD signal peak (a skewed Gaussian) using algorithms based on previous reports^29^. More than 430 single particles were identified in each sample and their ‘on’ intensities were binned with their noise levels, as shown in Fig. 5b,d. As expected, the ST-QDs exhibited widely different ‘on’ brightness levels; however, the observed 17-fold difference was smaller than that observed at the ensemble level. This may be the result of different *QY* when exciting at high fluence or due to the slightly prolate shape of the larger QDs, as alignment on the substrate may have an important contribution to excitation efficiency and emission polarization. Larger QDs also yielded a wider dispersion of brightness levels across the population, which was in accord with the dispersion in nanocrystal radius d*r*: derived from electron micrographs, as the volume dispersion scales with *r*^*2*^d*r*: Unlike the ST-QDs, the BE-QDs were very similar in histogrammed brightness levels, only differing by a slightly wider distribution for the Hg-rich QDs. We also compared these materials with commercially available Qdots composed of CdSe-based materials (see Supplementary Fig. 13 for details). Unfortunately, the characterized sizes and extinction coefficients of these materials were found to differ from what was given in specification information, so interpretation of the results is difficult. Importantly, for these single-particle studies, the average excitation rate per particle (with photon flux <10 mW cm^−2^) was orders of magnitude smaller than the saturated rate of fluorescent photon emission based on a typical excited state lifetime of QDs (∼20–50 ns), so saturation effects are not expected to have a significant role. However, lifetime differences will likely have a role in observed brightness when the populations approach saturation.

**Figure 1:**
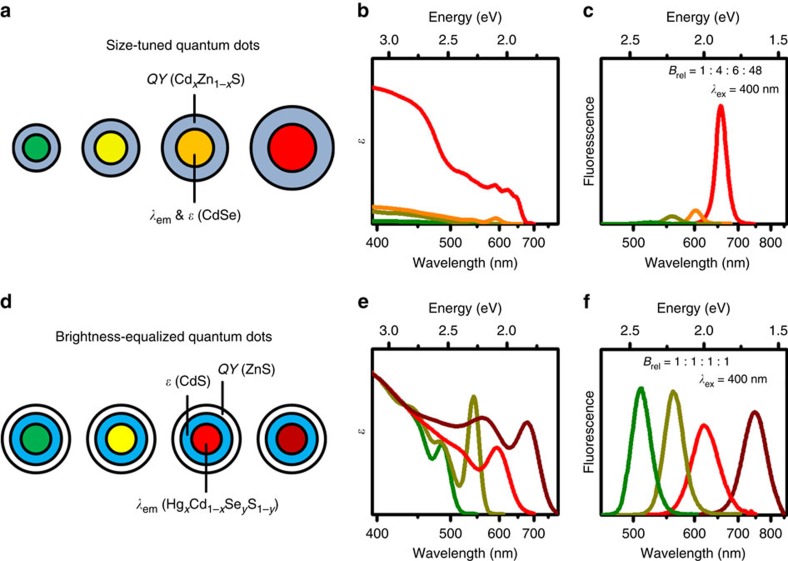
Comparisons between conventional size-tuned QDs (top) and brightness-equalized QDs (bottom). (**a**) Schematic depictions of ST-QD structures show that emission wavelength and extinction coefficient are largely dictated by the CdSe core size, and that the shell, composed of CdS, ZnS or alloys of the two, controls the quantum yield. (**b**) Extinction coefficient spectra of ST-QDs show a wide disparity in light absorption per-particle, resulting in (**c**) dissimilar fluorescence brightness values when excited at the same wavelength (here 400 nm). (**d**) Schematic depiction of BE-QD structures for which the core size is fixed and wavelength is tuned through the bandgap of composition-tunable alloys. The shell comprises two spherically concentric domains: the CdS shell is used to precisely match the extinction coefficients and the ZnS shell is used to equalize the quantum yields. (**e**) Extinction coefficient spectra of BE-QDs show convergence below 450 nm, resulting in (**f**) equalized fluorescence brightness when excited at the same wavelength (400 nm). Graphs depict representative experimental data plotted with wavelength axes scaled in proportion to energy.

**Figure 2:**
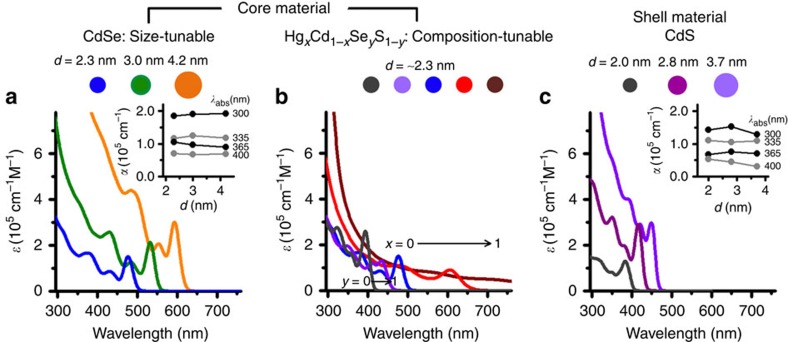
Extinction coefficient spectra of nanocrystalline materials used for cores and shells in this work. Spectra are shown for (**a**) CdSe QDs with different sizes, (**b**) Hg_*x*_Cd_1-*x*_Se_*y*_S_1-*y*_ QDs with fixed size (2.3 nm) and different compositions (*x*,*y*), and (**c**) CdS QDs with different core sizes. Insets show absorption coefficients at specific wavelengths.

**Figure 3:**
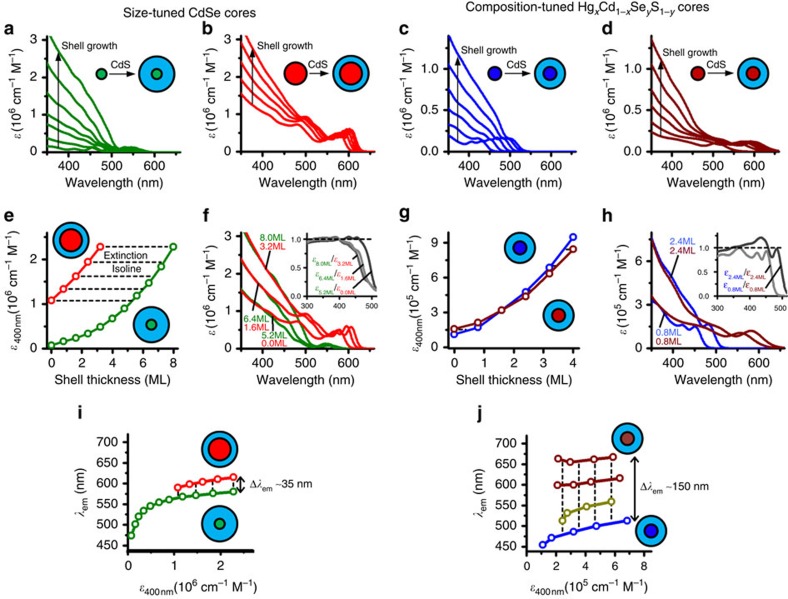
QD extinction coefficient equalization through CdS shell growth. The left two columns depict a representative equalization process for two CdSe cores with different sizes (2.0 and 4.3 nm) and the right two columns depict a representative equalization process for two alloy cores (Hg_*x*_Cd_1-*x*_Se_*y*_S_1-*y*_) with different compositions but similar sizes. (**a**–**d**) Extinction coefficient spectra are depicted for the four cores during capping with CdS in deposition increments of 0.8 monolayers; extinction increases with increasing shell thickness. Spectra depict the first 5–7 increments. (**e**,**g**) The trends in extinction coefficient values at 400 nm with different CdS shell thicknesses. Dashed lines are extinction isolines, connection points of equal extinction. (**f**,**h**) Spectra of two colours of QDs with matched extinction, showing strong correlation between 350 and 450 nm. Insets show ratios of spectra for each of these extinction-matched pairs. (**i**,**j**) The wavelength tunability of the resulting QDs, with substantially wider spectral range provided with alloy cores. Four example QD colours are shown in **j**.

**Figure 4:**
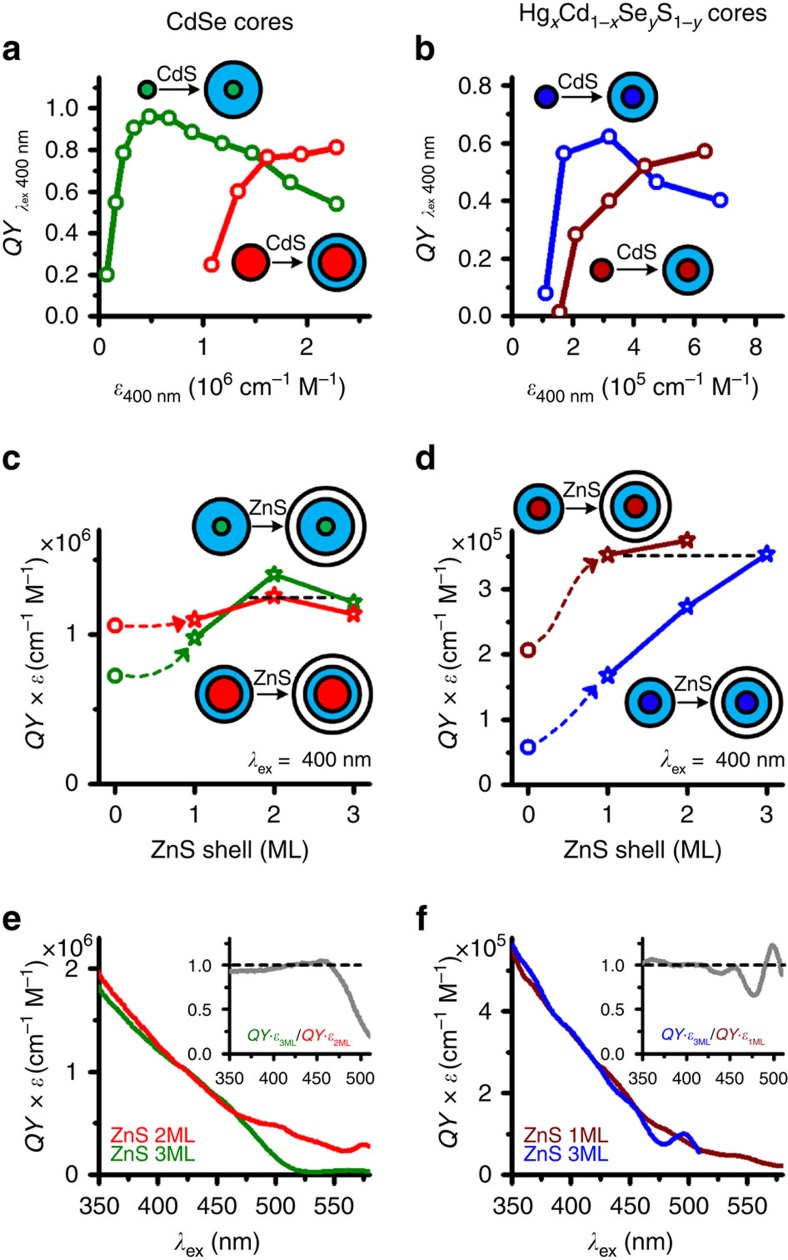
Quantum yield and brightness equalization. The left column depicts representative data for QDs based on size-tuned CdSe cores and the right column depicts representative data for composition-tuned cores. (**a**,**b**) Quantum yield values measured for QDs capped with different CdS shell thicknesses in organic solvents. The *x* axis is the extinction coefficient at 400 nm during shell growth and the *y* axis is *QY* with excitation at 400 nm. (**c**,**d**) Relative brightness measured for QDs capped with CdS and then ZnS shells with different ZnS shell thicknesses, with 400 nm excitation. (**e**,**f**) Relative brightness determined for different excitation wavelengths for two QD colours in aqueous solution. Insets show wavelength-dependent ratios of brightness.

**Figure 5:**
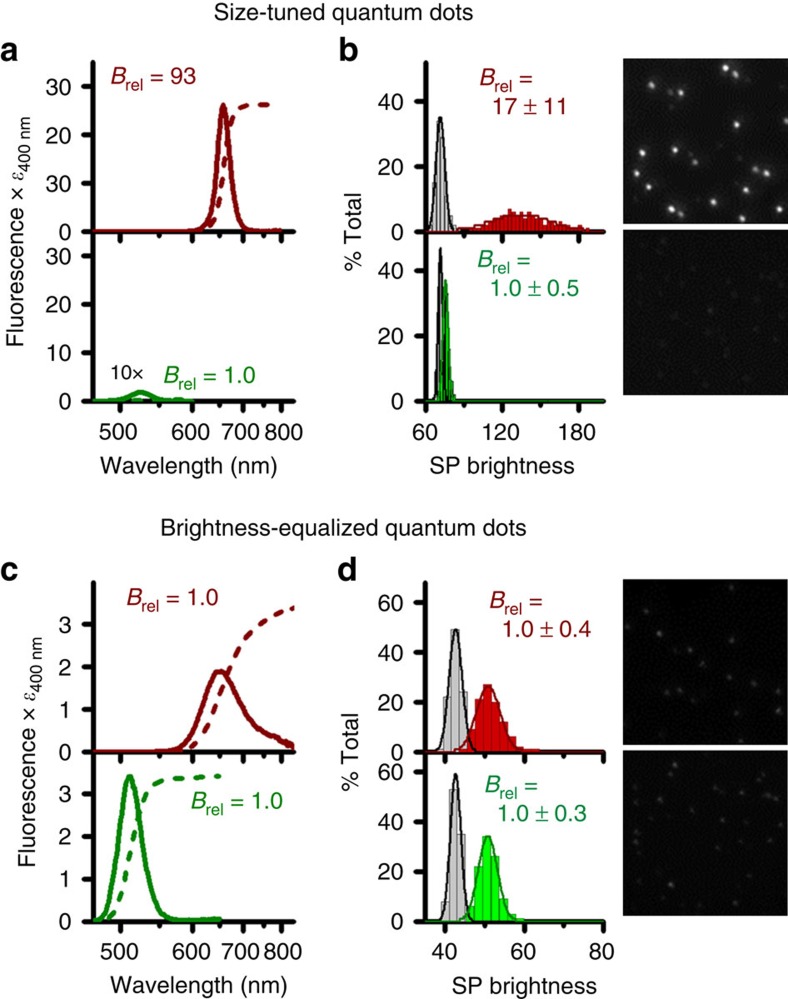
Brightness comparisons of ST-QDs and BE-QDs at the ensemble level and the single-particle level. (**a**) Measured fluorescence spectra and integrated brightness of two colours of conventional ST-QDs with 400 nm excitation. (**b**) Histogrammed single-particle (SP) brightness values of individual QDs measured using epifluorescence microscopy (images at right show example micrographs). (**c**) Measured fluorescence spectra and integrated brightness values of two colours of BE-QDs upon excitation at 400 nm. (**d**) Histogrammed brightness values of individual QDs measured using epifluorescence microscopy. Each fluorescence image has a square edge length of 14 μm.

For this demonstration, we prepared these particles to be as compact as possible (<6 nm) to maximize their utility for biomolecular sensing. Even smaller QDs could be prepared, but the brightness levels are not as precisely matched across as a wide range of excitation spectra; likewise, larger QD sets could also be prepared, with an advantage of greater absolute brightness and narrower emission bands. Notably, the bandwidth in energy is fairly uniform across different colours of BE-QDs (e.g., see Fig. 1f), as would be expected for QDs with similar core sizes and size dispersions. In contrast, the bandwidth in energy for ST-QDs decreases for more red-shifted particles because of decreasing confinement of the core material with increasing size. Further bandwidth engineering can be performed by choosing material compositions with different exciton sizes. Notably, BE-QD sizes were not precisely matched between different colours, as would be expected from their different compositions (see Fig. 2 for details) that require different quantities of material for extinction and QY balancing, although their size differences were much smaller than those of ST-QDs (see Supplementary Fig. 12 for details).

### Multiphoton brightness

**Figure 6:**
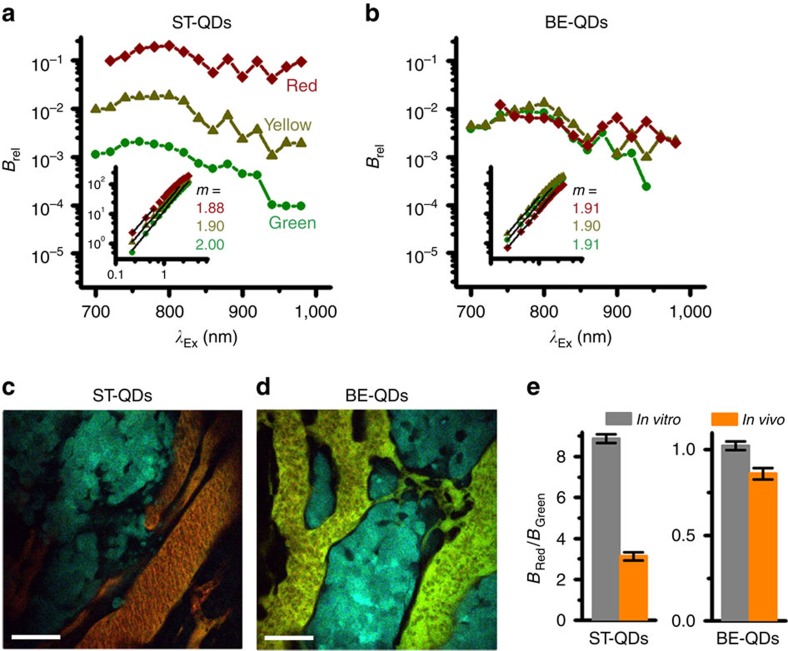
Brightness comparisons of ST-QDs and BE-QDs with two-photon excitation. (**a**) Measured brightness of three colours of conventional ST-QDs upon excitation at wavelengths between 700–1,000 nm. Inset shows fluorescence intensity (arbitrary units) versus excitation power (mW) plots for each QD with 780 nm excitation, with indicated log-log plot slopes (*m*). (**b**) Measured fluorescence brightness of three colours of BE-QDs upon excitation between 700 and 1,000 nm. Inset shows intensity-power plots. (**c**,**d**) Intravital multiphoton fluorescence images of a mouse mammary tumour showing fluorescence in blood vessels after intravenous injection of a mixture of green and red QDs in a 1:1 molar ratio. ST-QDs were injected into the mouse in **c**, *n*=3, and BE-QDs were injected into the mouse in **d**, *n*=3. Scale bar, 50 μm. Tumour cells expressing cyan fluorescent protein (CFP) provide contrast for interstitial tissue. (**e**) Measured brightness values of the red and green channels were divided for each *in vivo* experiment and plotted next to the corresponding ratio for *in vitro* values. Error bars denote standard error of measured brightness.

QDs are noted for being exceptionally bright multiphoton contrast agents because of extremely large multiphoton cross-sections^33^. Although the nonlinear photophysics underlying the multiphoton excitation of QDs are still not fully understood, it has been reported that the 2-photon absorption (2PA) cross-section of CdSe and CdTe nanocrystals scales roughly with *r*^4^ for 700–900 nm excitation^30^. Figure 6a shows the 2PA fluorescence brightness for three colours of ST-QDs excited across the 700–1,000 nm range using a femtosecond pulsed laser. The plot inset shows that the QDs exhibit power saturation curves with an exponent in the 1.88–2.00 range, consistent with non-saturating conditions. Note that the brightness axis is given in logarithmic scale: the relative brightness of the yellow QD is ∼30-fold greater than that of the green QD, where the curves are relatively flat (740–850 nm), and the red QDs are ∼220-fold brighter. At even longer wavelengths, this difference reaches ∼500-fold between red and green. In comparison, three BE-QDs have substantially closer brightness levels, as shown in Fig. 6b, with a brightness difference less than 1.6-fold in the 740–850 nm range.

To demonstrate the improved capacity for quantitative 2PA fluorescence imaging in a complex tissue, we prepared two sets of multicolour QDs. The ST-QD set comprised a red QD that had a greater brightness than the green QD. The BE-QD set had a similar brightness between the two colours. Importantly, the red QDs for both sets were identical so that they could serve as an internal control to allow the direct comparison between the green QDs, which had identical emission wavelengths, differing only in particle size and brightness (spectra are provided in Supplementary Fig. 14). We coated all QDs with the same polymeric coatings with a thick shell of polyethylene glycol (PEG) to mask disparities in size that could contribute to different circulation times in blood, then mixed the green and red QDs from each set together and injected each set intravenously into mice bearing orthotopic breast carcinomas (Polyoma middle T antigen-mouse mammary tumour virus). As shown in Fig. 6c,d, we imaged the vasculature of the tumours via intravital multiphoton fluorescence microscopy up to a depth of 50 μm, with an excitation wavelength of 780 nm, and measured the average brightness of the red and green channels for each QD set. We compared the relative red/green brightness ratio *in vivo* with the measured *in vitro* values (expected values). The ST-QD pair had a measured R/G brightness ratio of 3.13, but it was expected to be 8.87. The BE-QD pair had a measured R/G brightness ratio of 0.86, with an expected value of 1.02. These results show that the BE-QDs provide a substantial improvement in predicted photon output between different colours in comparison with ST-QDs. ST-QDs yield incorrect readings of *in vivo* concentration, in this experiment, by a factor of 2.8, whereas that difference is reduced to <1.2 in the case of the BE-QDs. The slight remaining difference from the expected values are likely due to differences in emitted light attenuation through the tissue and uncertain levels of QD saturation. The excitation power dependence onset of fluorescence saturation is much more widely dispersed for ST-QDs compared with BE-QDs (see insets in Fig. 6a,b), as red ST-QDs saturate at a much lower photon flux than green ST-QDs, whereas they have much better match across colours for BE-QDs.

## Discussion

In conclusion, we have demonstrated the ability to precisely tune the rate of photon absorption and photon emission of colloidal semiconductor QDs to balance their multicolour optical disparities. We created multicolour particles with nearly identical fluorescence brightness when excited at a wide range of excitation wavelengths (350–450 nm) that are compact in overall dimensions that can serve as next-generation emitters for quantitative imaging applications at the single-molecule level and in living systems. We project that these materials will be especially important for imaging in complex tissues where quantitative molecular imaging capabilities are significantly lacking, yielding a consistent and tunable number of photons per tagged biomolecule, for precise colour matching in light emitting devices and displays, and for photon-on-demand encryption applications. The same principles should be applicable to a wide variety of other materials for further expansion of the spectral range, including those with different crystal structures such as PbS and PbSe materials, III–V materials and other alloys.

## Methods

### Core QD synthesis

Binary CdSe and CdS and ternary CdSeS alloyed cores were synthesized from a non-injection heat-up synthesis using a cadmium carboxylate (cadmium behenate or cadmium myristate), SeO_2_ and elemental S as precursors and 1-octadecene (ODE) as solvent. In a typical synthesis, CdSe_*x*_S_1−*x*_ (0 ≤*x*≤ 1) QDs were synthesized by mixing Cd behenate (0.2 mmol), SeO_2_ (0.2*x* mmol) and S (0.2(1−*x*) mmol) in ODE (4 ml) at room temperature and heating to ∼230 °C at a rate of ∼20 °C min^−1^. The temperature was maintained at 230 °C for ∼15 min and the reaction was quenched by decreasing the temperature to ∼100 °C and diluting with chloroform (10 ml) containing oleylamine (OLA; 0.6 ml) and oleic acid (1 ml). Finally, a mixture of acetone and methanol was added to precipitate the pure cores. Alloyed HgCdSe(S) cores were prepared via mercury cation exchange on CdSe(S) cores. Typically, CdSe cores dispersed in oleylamine were heated to 50–150 °C and mixed with mercury octanethiolate (Cd/Hg=1:2) to induce cation exchange. After a desired amount of redshift was observed in the absorption spectrum, the reaction was quenched by precipitating the particles with a mixture of acetone and methanol. Details on the chemicals and synthetic parameters for cores with different sizes and compositions are provided in Supplementary Notes 1 and 2.

### CdS and ZnS shell growth

Both CdS and ZnS shells were grown following conventional layer-by-layer shell growth protocols used in core/shell QD synthesis. Cadmium oleate in ODE–decylamine–trioctyphosphine (TOP), zinc acetate in OLA and hexamethyldisilathiane ((TMS)_2_S) in TOP were used as shell precursors for Cd, Zn and S, respectively. For the first ML of CdS shell growth on HgCdSe(S) cores, Cd and S precursors without TOP (Cd oleate in ODE–decylamine and (TMS)_2_S in ODE), were used because TOP can degrade bare HgCdSe(S) cores due to the strong binding affinity of TOP to Hg ions. In a typical synthesis, 50–100 nmol of pure core QDs dispersed in ODE/OLA solvent (2:1 (v/v)) were heated to the desired temperature for the shell growth: 120–190 °C for CdS shell growth depending on the core size and the shell thickness and ∼190 °C for ZnS shell growth. In each layer-by-layer shell growth cycle, the S precursor was added dropwise and allowed to react for 15–20 min, and then the Cd or Zn precursor was added dropwise and allowed to react for 15–20 min. To prevent homogeneous nucleation of shell materials, the CdS shell was grown with 0.8 ML increments (80% of total precursors needed to grow 1 ML of shell) per cycle instead of 1 ML. The ZnS shell was grown in 0.5 ML steps so that two cycles of S–Zn addition were needed to grow 1 ML. The quantities of precursors needed to grow each ML were calculated using the single ML thickness of ∼0.3 nm, which is the thickness of one CdS layer along the (100) lattice direction of the zinc-blende CdS crystal. A precisely measured aliquot (200 μl) was withdrawn and diluted 10 × in chloroform after each shell growth cycle to monitor the extinction coefficient increase. The reaction was quenched by decreasing the temperature and precipitating the nanocrystals with acetone. Detailed reaction conditions for shell growth are provided in Supplementary Note 3.

### QD phase transfer

Core/shell QDs were transferred to water by coating with an amphiphilic polymer (amphipol, 40% octylamine-conjugated polyacrylic acid, MW=∼2,900). Typically, purified core/shell QDs dispersed in chloroform (∼1 nmol ml^−1^, 2–10 ml) were mixed with 2,000–2,500 × molar excess amphipol. Then chloroform was slowly evaporated under vacuum while vigorously stirring the mixture. After removing chloroform completely, 10 mM NaOH solution in distilled water (2–3 ml nmol^−1^ of QD) was added and stirred for several hours until the amphipol-coated QDs were fully dispersed. Finally, the solution was centrifuged to remove any QD aggregates. For PEG conjugation, amphipol-coated QDs were dispersed into PBS and further purified using size-exclusion chromatography and dialysis to remove free amphipol polymers. The carboxylic acid groups on the surface of amphipol-coated QDs were reacted with 40,000 × molar excess of monoamino-PEG (750 Da) in PBS using 4-(4,6-dimethoxy-1,3,5-triazin-2-yl)-4-methylmorpholinium chloride as the coupling reagent. Finally, PEG-coated QDs were purified by dialysis to remove excess amino-PEG and other impurities. More details on QD phase transfer are provided in Supplementary Note 4.

### Instrumentation

Absorption spectra were obtained using an Agilent Cary 5000 UV-Vis-NIR spectrophotometer (Agilent). Fluorescence and photoluminescence excitation spectroscopy were obtained with a Horiba NanoLog spectrofluorometer (Horiba). TEM images were acquired using a JEOL 2010 LaB_6_ high-resolution microscope (JEOL). ICP-OES was performed using a PerkinElmer Optima 2000DV instrument (PerkinElmer). Single-particle fluorescence microscopy was performed using a Zeiss Axio Observer.Z1 inverted microscope (Zeiss) with a 100 × , 1.45 NA Plan-Fluar objective with halogen lamp illumination. *In vitro* multiphoton fluorescence measurements were performed using a Zeiss 710 confocal scanning Axio Observer.Z1 inverted microscope (Zeiss) with a 10 × , 0.30 NA Plan-Neofluar objective with tunable Mai-Tai Ti-Sapphire laser excitation. Additional details are provided in Supplementary Note 5.

### Extinction coefficient measurements of cores

Extinction coefficients, *ε* (cm^−1^ M^−1^), of CdSe, CdS and CdSeS cores were measured using the Beer–Lambert law of absorbance:




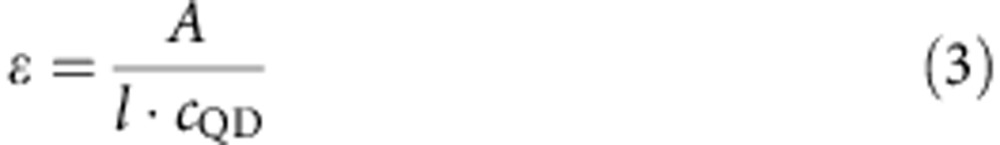




where *A* is the absorbance (unitless), *l* is the path length (cm) of the cuvette and *c*_QD_ is the concentration of QDs (M). *A* of a core solution was measured using an ultraviolet–visible absorption spectrophotometer. Typically, a 0.5-cm path length cuvette (*l*=0.5 cm) was used. *c*_QD_ was calculated by combining information from two independent measurements: the average particle radius, *r* (nm), from TEM and elemental concentration of Cd in solution, *c*_Cd_ (M), from ICP-OES. The average number of Cd atoms per core QD, *n*_Cd_, was calculated using:




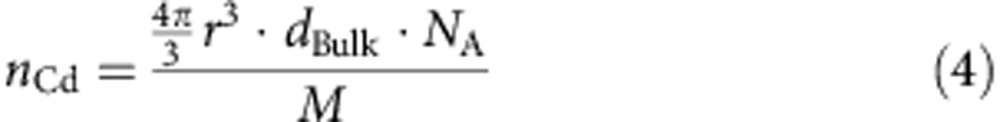




where *M* is the molecular weight of the material and *N*_A_ is the Avogadro constant (6.022 × 10^23^ mol^−1^), based on the assumption that all particles are spherical and have the same density as the bulk material, *d*_Bulk_. Then *c*_QD_ can be calculated from both *c*_Cd_ and *n*_Cd_ as




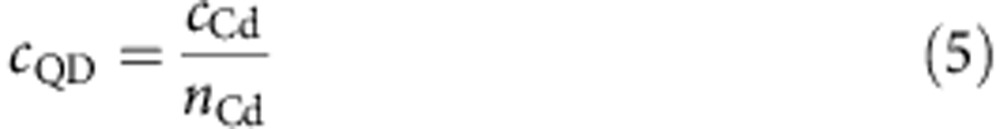




The value of *ε* for HgCdSe(S) alloy cores was acquired by carefully measuring the difference in the absorption spectra before and after mercury cation exchange, based on the assumption that the total number of particles was conserved during the reaction. Additional information on the ICP-OES analysis is provided in Supplementary Note 5, and detailed descriptions for calculations are provided in Supplementary Note 6.

### QY measurements

For fluorescence *QY* measurements, a dilute QD sample (absorbance ∼0.05 at 491 nm) was prepared and its absorption, fluorescence and photoluminescence excitation spectra were acquired. The same set of spectra were acquired for a reference (fluorescein in 0.1 M NaOH, *QY*=92%). The relative *QY* was calculated using the following equation.




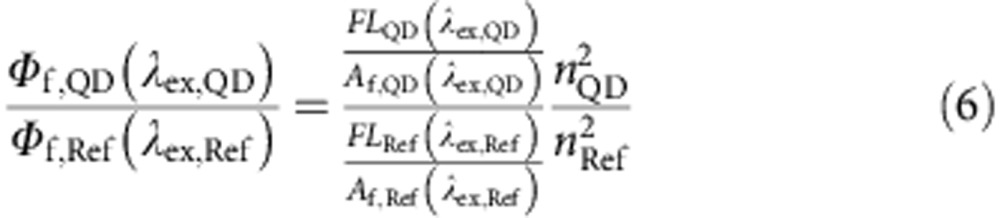




where *Φ*_f,_ is the fluorescence *QY*, *FL* is the total fluorescence intensity (the integrated area of the emission spectrum in wavelength scale) with excitation at *λ*_ex_ normalized by the intensity of the excitation light, *A*_f_ is the absorption factor (or absorptance) at *λ*_ex_ and *n* is the refractive index of the solvent. *A*_f_ is the fraction of incident light that is absorbed by the sample which is expressed as




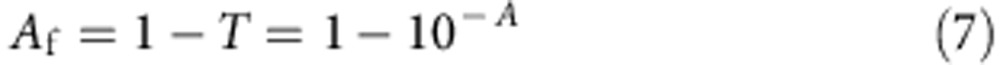




where *T* and *A* are the transmittance and the absorbance, respectively. It should be noted that *A*_f_ is proportional to the number of photons absorbed by the sample, whereas *A* is a logarithmic ratio. Therefore, *A*_f_ should be used to for accurate calculation of relative *QY*, not *A*. Using *A*_f_ instead of *A* is especially important for excitation wavelength-dependent QY calculations when the excitation wavelength used for QDs and the reference are different. Additional details on *QY* calculations are described in Supplementary Note 7.

### Single-particle brightness measurements

Polymer-coated QDs dispersed in PBS were spin-coated on glass coverslips and imaged via epifluorescence microscopy to acquire single-particle fluorescence movies. The movies were analysed using algorithms based on previous reports^29^ that are described in detail in Supplementary Note 8.

### Intravital microscopy

All procedures involving animals were conducted in accordance with the National Institutes of Health regulations and approved by the Albert Einstein College of Medicine Animal Use Committee. Tumour tissue from female FVB mice transgenically expressing Polyoma middle T antigen under direction of the mouse mammary tumour virus promoter was cut into pieces of 2–3 mm and coated in Matrigel. One piece of tumour was surgically implanted in the right lower mammary fat pad of a non-transgenic FVB mouse and allowed to grow to ∼1 cm in diameter over 4–6 weeks. Intravital imaging of each tumour-bearing mouse was performed using a custom-built multiphoton microscope Olympus IX-71 (Olympus) with a 20 × , 0.95 NA water immersion objective with tunable femtosecond Mai-Tai laser tuned to 780 nm. Fluorescence and second-harmonic signals were separated via dichroic mirrors and collected using separate photomultiplier tubes. Details on the animal model, microscopy technique and image analysis are provided in Supplementary Note 9.

## Additional information

**How to cite this article:** Lim, S. J. *et al*. Brightness-equalized quantum dots. *Nat. Commun*. 6:8210 doi: 10.1038/ncomms9210 (2015).

## Supplementary Material

Supplementary Information Supplementary Figures 1-14, Supplementary Tables 1-2, Supplementary Notes 1-9 and Supplementary References.
