# Supplementary material for: Brightness-equalized quantum dots
Source: Nat Commun. 2015 Oct 5;6:8210. doi: 10.1038/ncomms9210 (PMC4594210; doi:10.1038/ncomms9210)
Supplement: Supplementary Information — Supplementary Figures 1-14, Supplementary Tables 1-2, Supplementary Notes 1-9 and Supplementary References. [file ncomms9210-s1.pdf]

Supplementary Information

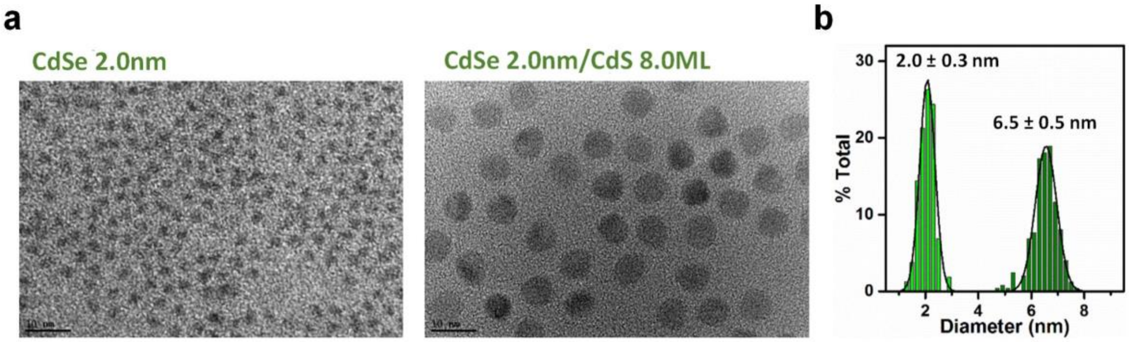

Supplementary Figure 1. Transmission electron microscopy of core CdSe nanocrystals and core/shell CdSe/CdS nanocrystals. (a) TEM images of 2.0 nm CdSe nanocrystals before (left) and after (right) deposition of 8.0 monolayers of CdS shell. (b) Histograms of nanocrystal diameter. Scale bars: 10 nm.

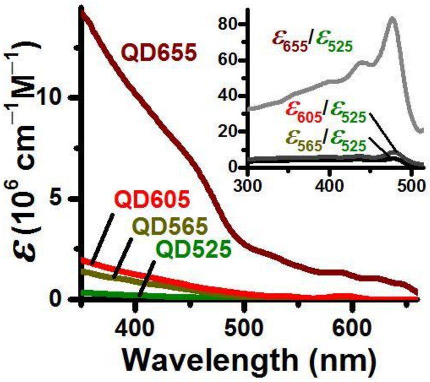

Supplementary Figure 2. Extinction coefficient spectra of four colors of CdSe/CdZnS QDs, demonstrating a large difference depending on size/color. The inset shows the relative differences, obtained by dividing each spectrum by the QD525 spectrum.

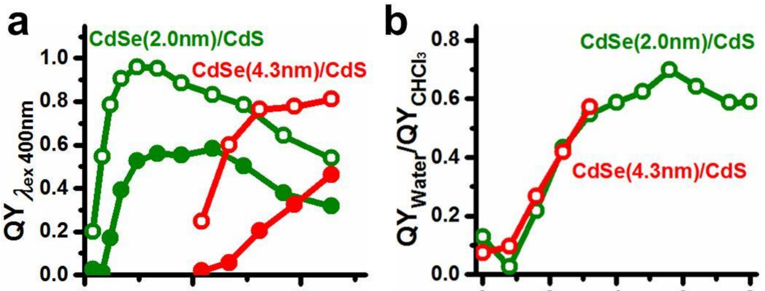

Supplementary Figure 3. Quantum yield data for core/shell CdSe/CdS nanocrystals, comparing values in nonpolar solvents (open circles) and after phase transfer to aqueous solution (closed circles). (a) All QDs exhibited a substantial decrease in quantum yield after phase transfer, but this difference decreased with increasing shell thickness. (b) The relative quantum yield in nonpolar solvents and polar solvents correlated with the number of monolayers deposited, requiring at least 3.2 monolayers to provide consistently high quantum yield.

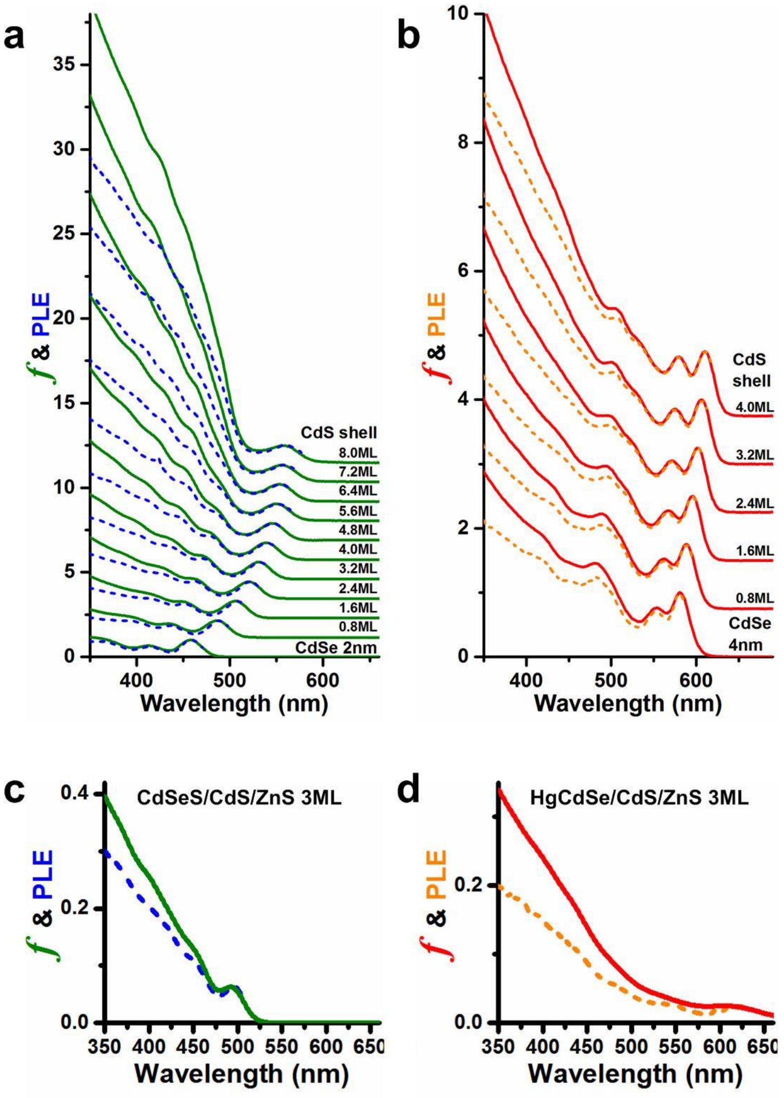

Supplementary Figure 4. Comparison between absorption factor (*f*) spectra (solid lines) and photoluminescence excitation (PLE) spectra (dashed lines) for QDs capped with different shell thicknesses. (a) Trends in absorption factor spectra and PLE spectra for CdSe/CdS nanocrystals with 2.0 nm cores and a variety of CdS shell thicknesses up to 8.0 ML. (b) Trends in absorption factor spectra and PLE spectra for CdSe/CdS nanocrystals with 4.3 nm cores and a variety of CdS shell thicknesses up to 4.0 ML. (c) Absorption factor and PLE spectra for CdSeS/CdS/ZnS nanocrystals after deposition of 3.0 ML of ZnS shell. (d) Absorption factor and PLE spectra for HgCdSe/CdS/ZnS nanocrystals after deposition of 3.0 ML of ZnS shell. Deviations between the absorption factor and PLE spectra are largely due to slight differences in quantum yield at different wavelengths, as discussed in detail in Supplementary Reference 1.

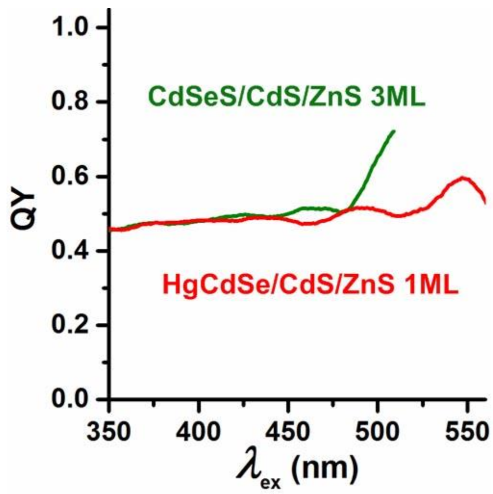

Supplementary Figure 5. Wavelength-dependent quantum yield measurements for brightness-equalized CdSe/CdS/ZnS nanocrystals and HgCdSe/CdS/ZnS after transfer to water.

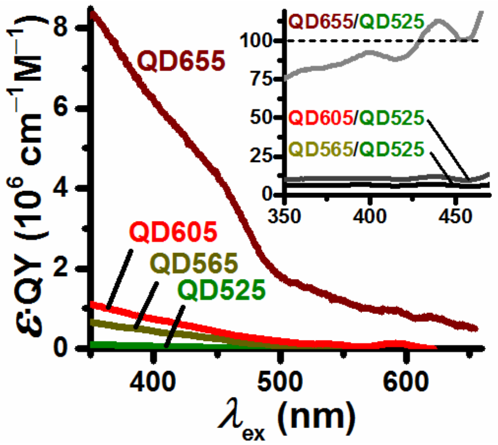

Supplementary Figure 6. Brightness spectra of 4 colors of ST-QDs across a range of excitation wavelengths from 350-650 nm. The inset shows the relative differences, obtained by dividing each spectrum by the QD525 spectrum.

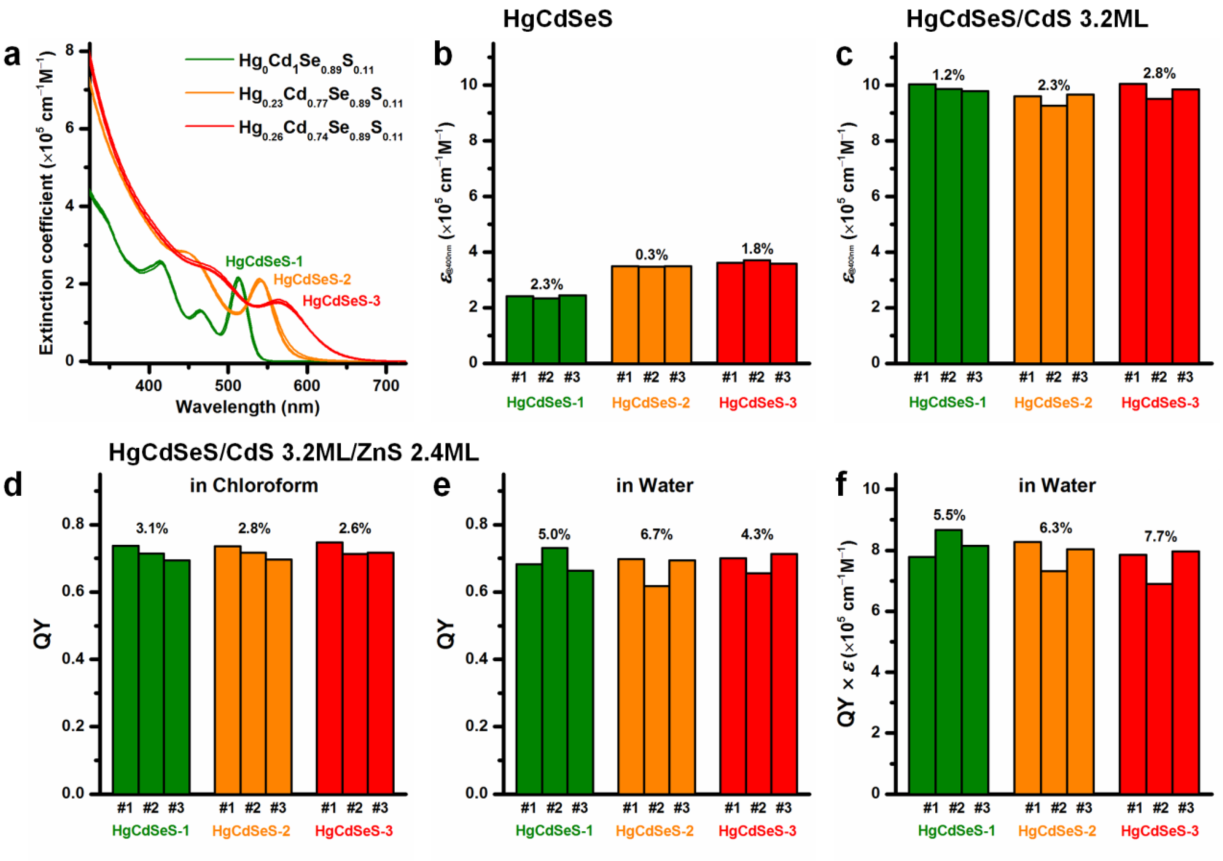

Supplementary Figure 7. Batch-to-batch variation in the optical properties of brightness-equalized QDs during synthesis. (a) Absorption spectra of the three batches of $Hg_xCd_{1-x}Se_yS_{1-y}$ alloy QDs with three different compositions (green: HgCdSeS-1, x=0, y=~0.89; orange: HgCdSeS-2, x=~0.23, y=~0.89; red: HgCdSeS-3, x=~0.26, y=~0.89). (b, c) Batch-to-batch variation in extinction coefficient at 400 nm ($\varepsilon_{@400nm}$) of HgCdSeS alloy cores (b) and HgCdSeS/CdS 3.2ML extinction-matched core/shell QDs (c). (d, e) Batch-to-batch variation in the quantum yield of HgCdSeS/CdS 3.2ML/ZnS 2.4ML brightness-equalized QDs in chloroform (d) and in water (e). (f) Batch-to-batch variation in the relative brightness (QY × ε) of brightness-equalized QDs in water.

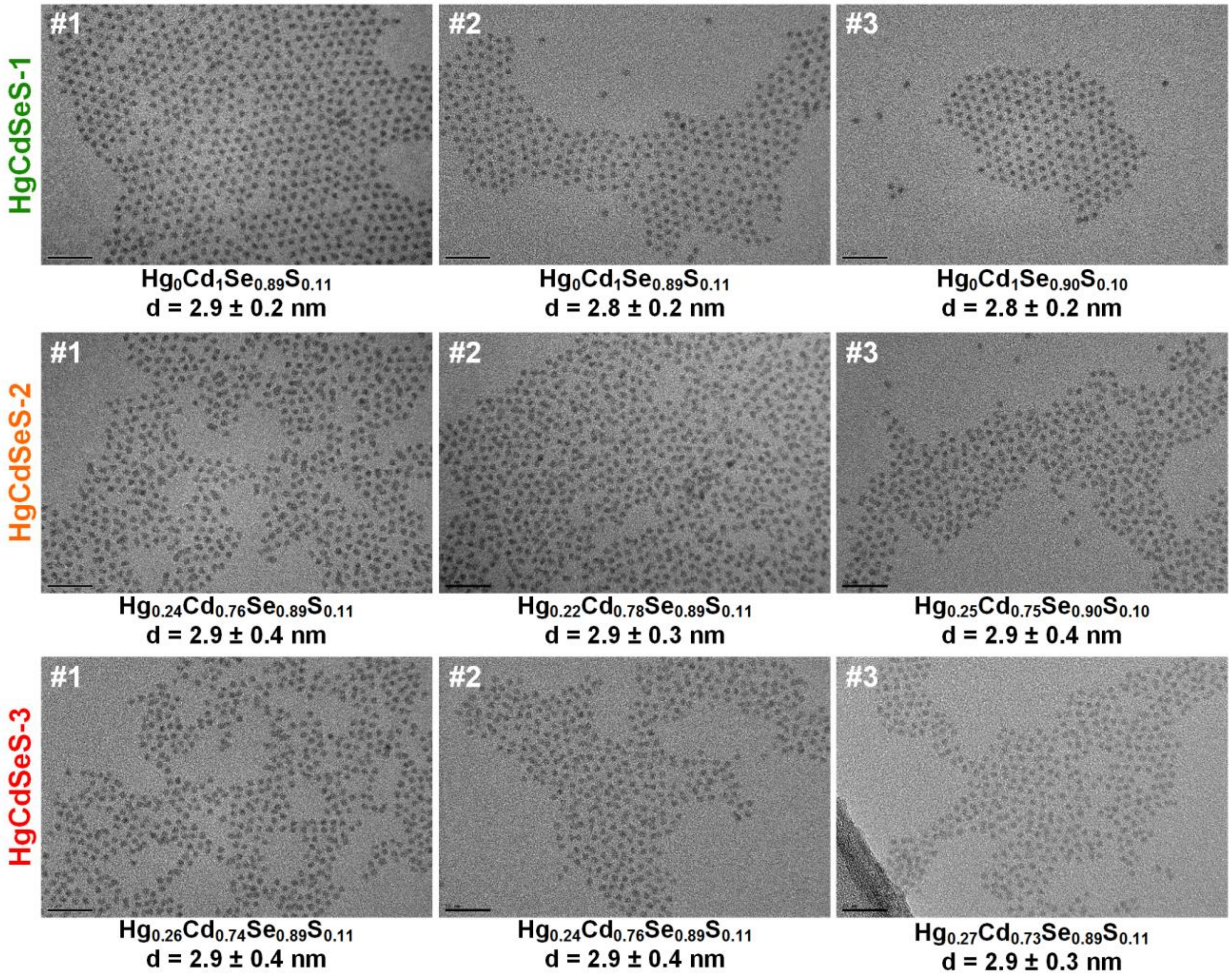

Supplementary Figure 8. Batch-to-batch variation in the size and composition of HgCdSeS alloy core QDs. TEM images of the three batches (#1–3) of $Hg_xCd_{1-x}Se_yS_{1-y}$ alloy QDs with three different compositions: HgCdSeS-1 (x=0, y=~0.89; top row), HgCdSeS-2 (x=0.22–0.25, y=~0.89; bottom row), and HgCdSeS-3 (x=0.24–0.27, y=~0.89; bottom row). Atomic compositions were obtained via elemental analysis. Scale bars: 20 nm.

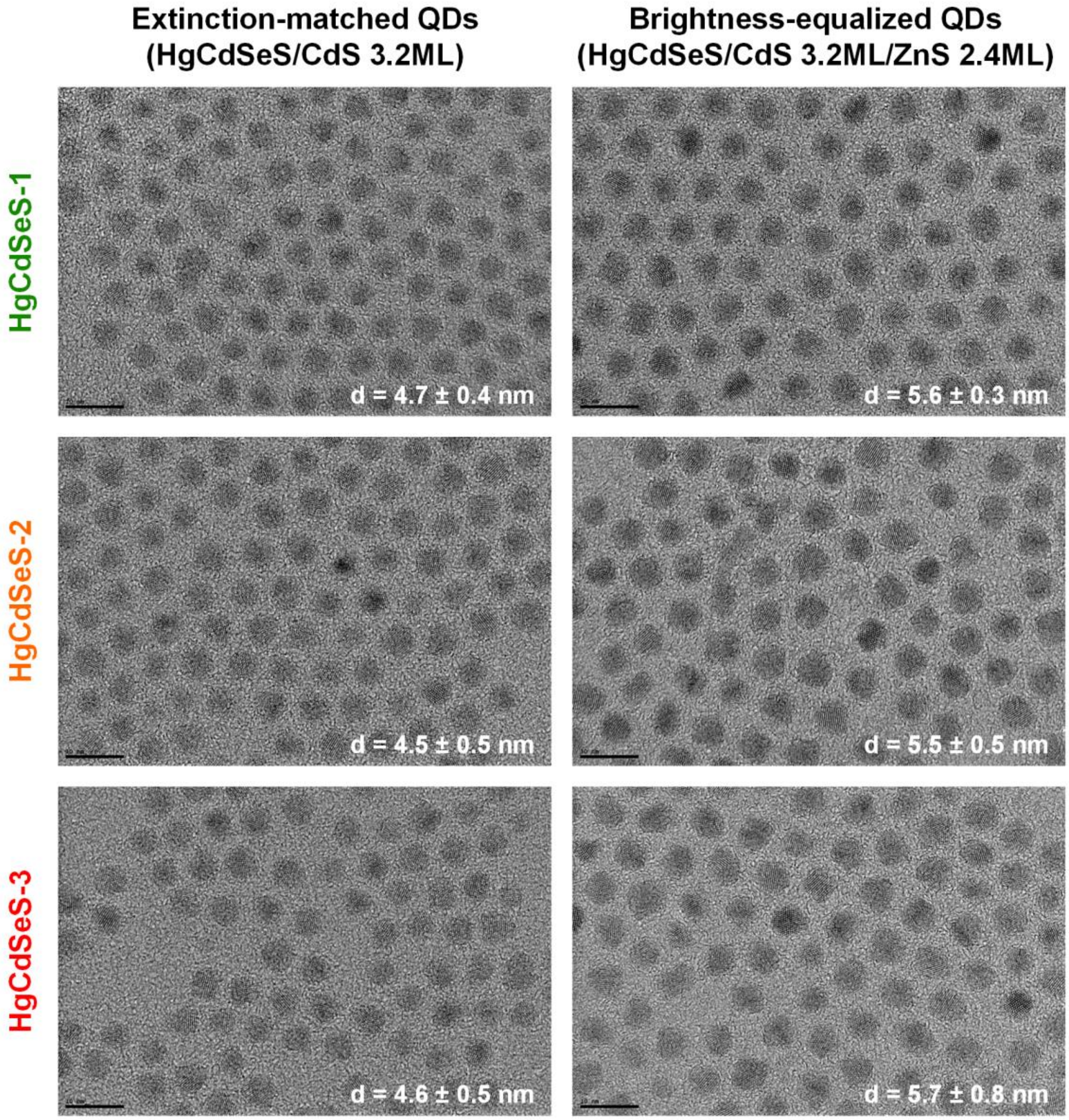

Supplementary Figure 9. TEM images of representative examples of the extinction-matched (left) and the brightness-equalized QDs (right) made from HgCdSeS-1 ($Hg_0Cd_1Se_{0.89}S_{0.11}$, top), HgCdSeS-2 ($Hg_{0.24}Cd_{0.76}Se_{0.89}S_{0.11}$, middle), and HgCdSeS-3 ($Hg_{0.26}Cd_{0.74}Se_{0.89}S_{0.11}$, bottom). Scale bars: 10 nm.

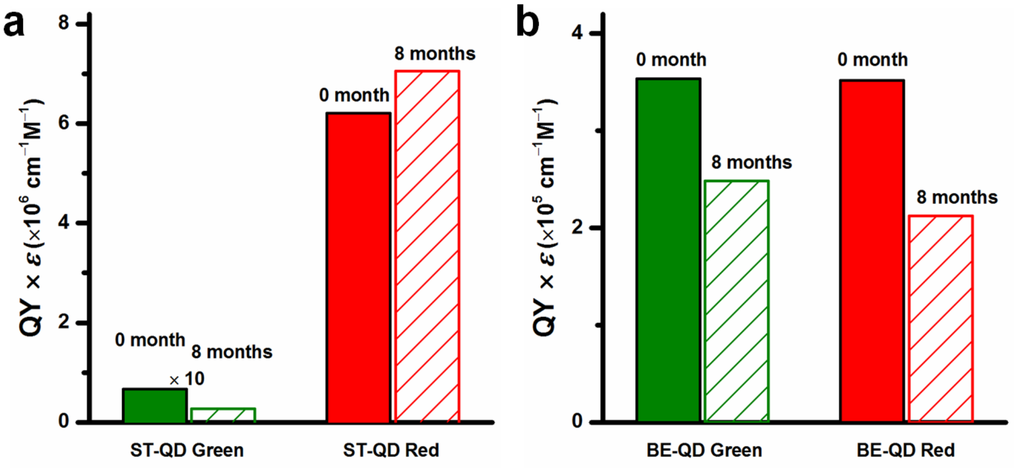

Supplementary Figure 10. Long-term changes in the brightness of (a) size-tuned (ST) QDs and (b) brightness-equalized (BE) QDs.

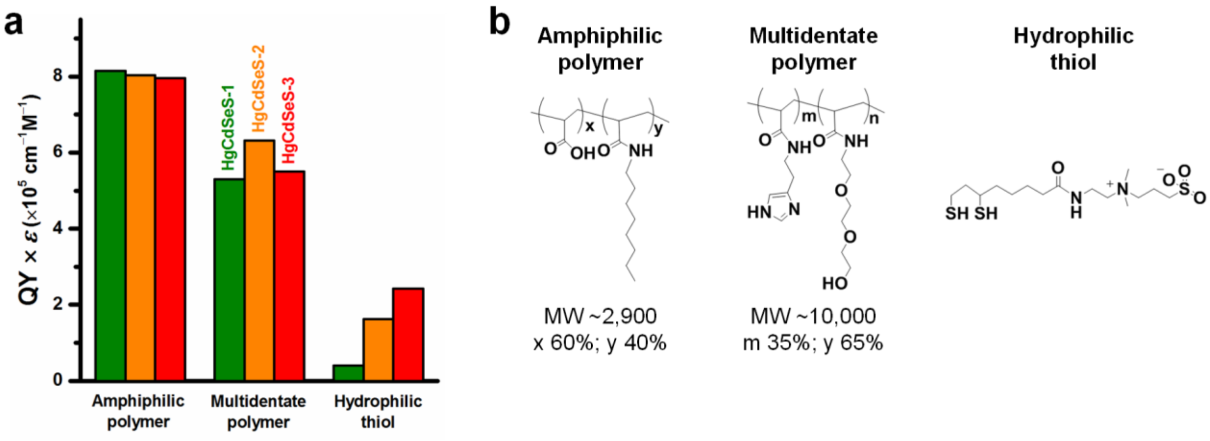

Supplementary Figure 11. Ligand-dependent brightness (QY × ε) of BE-QDs. (a) Relative brightness of BE-QDs coated with an amphiphilic polymer (left), multidentate polymer (middle), and hydrophilic thiol ligand (right). (b) Molecular structures of the different coating types.

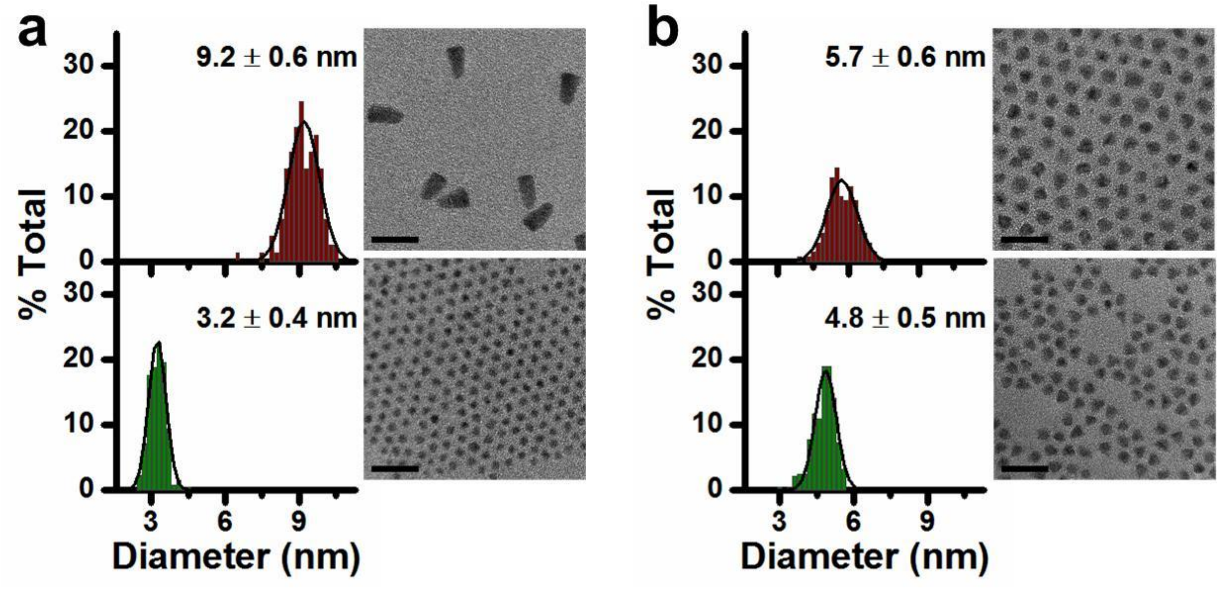

Supplementary Figure 12. Histograms of nanocrystals sizes for nanocrystals in Figure 5 in the main text. (a) ST-QDs with red (top) and green (bottom) emission. (b) BE-QDs with red (top) and green (bottom) emission. Scale bars: 10 nm.

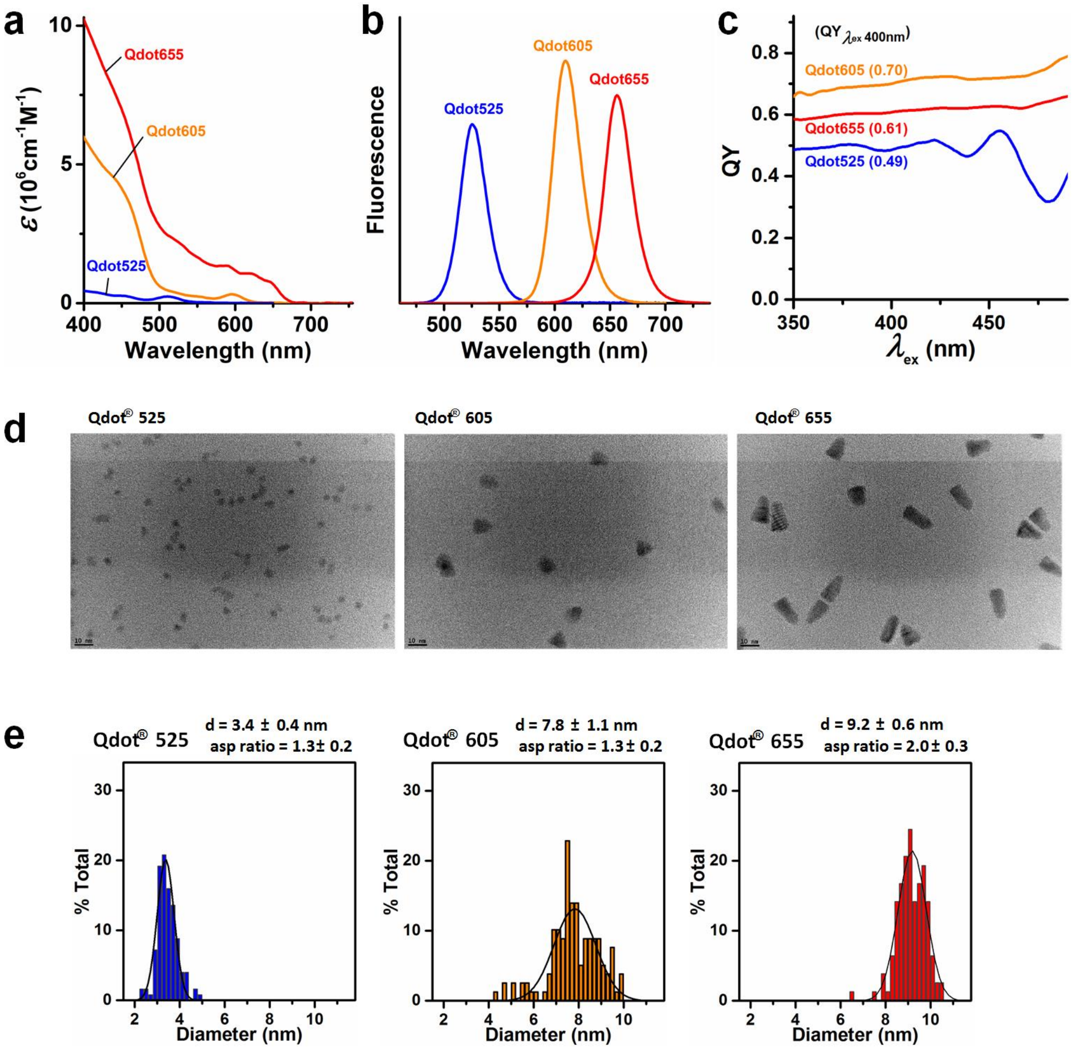

Supplementary Figure 13. Optical and structural characterization of commercial QDs (Streptavidin-Qdot® from Life Technologies). (a) Extinction coefficient spectra of 3 colors of commercial QDs. Measurements were based on the QD concentration provided in the product specifications. The extinction coefficient of Qdot®605 was significantly different from the value provided in the specifications. (b, c) Fluorescence spectra and excitation wavelength-dependent quantum yields of commercial QDs. Numbers in parenthesis in (c) represents the QY with excitation at 400 nm. (d, e) TEM images and size histograms of commercial QDs, showing large size differences between the QDs with different colors. Scale bars: 10 nm.

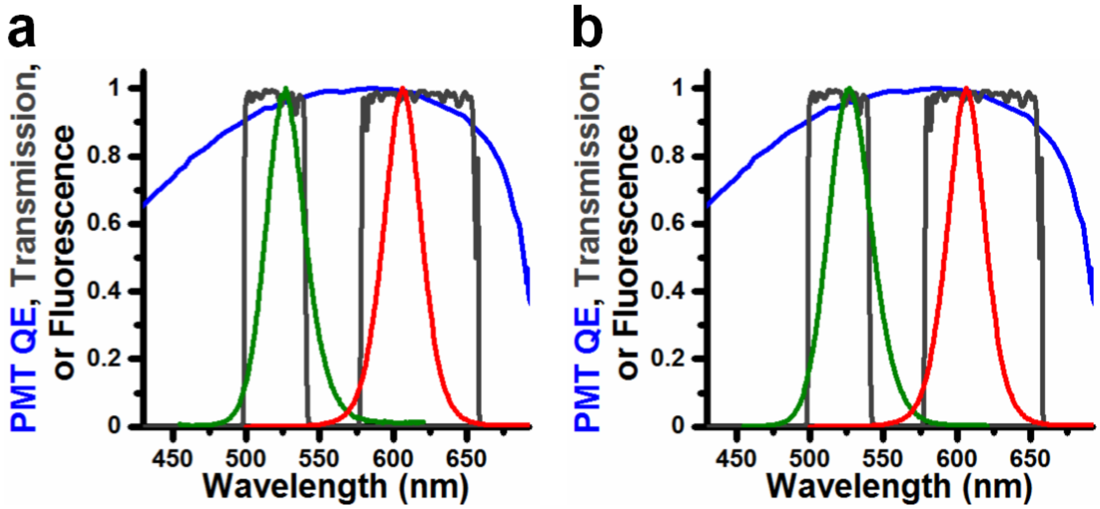

Supplementary Figure 14. Detector spectral quantum efficiency (QE) curve, filter transmission bands, and QD emission spectra for *in vivo* multiphoton confocal measurements with (a) size-tuned green and red QDs and (b) brightness-equalized green and red QDs.

Supplementary Table 1. Synthetic conditions for W CdSe QDs

QD diameter	Se injection temperature	DPP/TOP stock	Growth time	Note
2.0 nm	300°C	100 μL	0 s	Heating mantle was removed right before the Se precursor injection. Immediately after the Se injection, the solution was rapidly cooled down to ~200°C in ~1 min using a stream of air to quench particle growth.
2.4 nm	325°C	100 μL	0 s	
4.3 nm	365°C	10 μL	50 s	Se precursor was injected with the heating mantle attached. In 50s, the heating mantle was removed and the solution was rapidly cooled under a stream of air.

Supplementary Table 2. Synthetic conditions for HgCdSeS cores

CdSeS QD diameter (amount)	Hg precursor (amount)	Solvent (amount)	Temp. (°C)	Reaction time	λ_{Abs} (CdSeS)	λ_{Abs} (HgCdSeS)
2.3 nm (CdSe) (100 nmol)	$Hg(OT)_2$ (24 μmol)	OLA (5 mL)	50	15 min	481 nm	[a] 520 nm
2.3 nm (CdSe) (100 nmol)	$Hg(OT)_2$ (24 μmol)	OLA (5 mL)	100	120 min	481 nm	[b] 565 nm
2.3 nm (CdSe) (100 nmol)	$Hg(Ac)_2$ (36 μmol)	$CHCl_3$ (5 mL)	r.t.	30 min	481 nm	[c] ~730 nm
3.0 nm (CdSe) (100 nmol)	$Hg(Ac)_2$ (78 μmol)	$CHCl_3$ (10 mL)	r.t.	10 min	532 nm	[d] 690 nm
2.9 nm ($CdSe_{0.89}S_{0.11}$) (100 nmol)	$Hg(OT)_2$ (48 μmol)	OLA (5 mL)	40	30 min	513 nm	[e] 541–543 nm
2.9 nm ($CdSe_{0.89}S_{0.11}$) (100 nmol)	$Hg(OT)_2$ (48 μmol)	OLA (5 mL)	120	55 min	513 nm	[f] 562–564 nm

[a,b]HgCdSeS cores in Fig. 3; [c]HgCdSeS (x=0-1) core in Fig. 2b; [d] HgCdSe core for QD750 in Fig. 1e,f; [e] HgCdSeS-2 in Supplementary Figures 7–9; [f] HgCdSeS-3 in Supplementary Figures 7–9. r.t. = room temperature.

Supplementary Notes

Supplementary Note 1: Chemicals

Commercial sources. Cadmium oxide (CdO, 99.99+%), cadmium acetate hydrate ($Cd(Ac)_2 \cdot H_2O$, 99.99+%), mercury acetate ($Hg(Ac)_2$, 99.999%), diethylzinc solution ($Zn(Et)_2$, 1.0 M in hexane), selenium dioxide (SeO_2, ≥99.9%), selenium powder (Se, ~100 mesh, 99.99%), sulfur powder (S, 99.98%), hexamethyldisilathiane ($(TMS)_2S$, synthesis grade), 2,2'-dithiobis(benzothiazole) (99%), octanethiol (OT, ≥98.5%), tributylphosphine (TBP, 97%), diphenylphosphine (DPP, 98%), 1,2-hexadecanediol (HDD, 97%), N-methylformamide (NMF, ≥99%), tetramethylammonium hydroxide solution (TMAH, 25 wt.% in methanol), and fluorescein isothiocyanate isomer I (fluorescein, ≥90%) were purchased from Sigma-Aldrich. Cadmium chloride anhydrous ($CdCl_2$, 99.99%), and zinc acetate ($Zn(Ac)_2$, 99.98%) were obtained from Alfa Aesar. 1-octadecene (ODE, 90% tech.), oleylamine (OLA, 80–90% C18-content), decylamine (DA, 99%), oleic acid (OAc, 90% tech.), myristic acid (MAc, 99%), and 4-(4,6-dimethoxy[1,3,5]triazin-2-yl)-4-methylmopholinium chloride (DMTMM) were purchased from Acros Organics. Behenic acid (BAc, 99%) was obtained from MP Biomedicals and octadecylphosphonic acid (ODPA, >99%) was purchased from PCI Synthesis. Trioctylphosphine oxide (TOPO, 99%) and trioctylphosphine (TOP, 97%) were acquired from Strem Chemicals. 750 Da monoamino-polyethylene glycol (amino-PEG) was purchased from Rapp Polymere. Solvents including chloroform, hexane, toluene, methanol, acetone were purchased from various suppliers including Acros Organics, Fisher Scientific, Macron Fine Chemicals. Streptavidin-Qdot® conjugate kit including Qdot®655 was purchased from Life Technologies. All chemicals above were used as purchased.

Cadmium behenate ($Cd(BAc)_2$) and cadmium myristate ($Cd(MAc)_2$) synthesis. $Cd(BAc)_2$ was prepared using literature methods.[2,3] $CdCl_2$ (5 mmol) was dissolved in methanol (200 mL), filtered to remove any undissolved debris, and transferred to a 500-mL dropping funnel. BAc (15 mmol) was dissolved in a mixed solvent of methanol (1.25 L) and chloroform (150 mL) with the addition of TMAH (25% wt. in methanol, ~8 mL). The mixture was stirred for >1 h until complete dissolution of the white BAc powder, and the solution was filtered to yield a clear, colorless solution. The $CdCl_2$ solution was added dropwise to the BAc solution with vigorous stirring in a 2-L beaker. The entire $CdCl_2$ solution was added in ~1 h and the mixture was left stirring for an additional 1 h. $Cd(BAc)_2$ was collected by vacuum filtration and washed three times with methanol (150–200 mL per wash) on a filter funnel. The product was dried on the funnel for several hours and then dried under vacuum at ~50°C overnight. $Cd(MAc)_2$ was synthesized using the same process except BAc was replaced with MAc and chloroform was not used to dissolve MAc.

$Hg(OT)_2$ synthesis: $Hg(OT)_2$ was synthesized by following literature protocols.[4] Briefly, $Hg(Ac)_2$ (2 mmol) was dissolved in methanol (100 mL) and filtered. OT (6 mmol) was mixed with methanol (1 L) and KOH (6 mmol). $Hg(Ac)_2$ solution was added dropwise to the OT solution while vigorously stirring to produce a white $Hg(OT)_2$ precipitate. $Hg(OT)_2$ was collected by vacuum filtration and washed multiple times with methanol, and once with ether. The product was dried overnight under vacuum.

40% octylamine-modified polyacrylic acid (amphiphol) synthesis: Amphipol was synthesized and purified using methods described in the literature.[5] M.W. ~2,911.

Multidentate polyimidazole ligand synthesis: A polyimidazole ligand was synthesized using similar methods from the literature.[6-7]

Zwitterionic bidentate thiol ligand synthesis: The thiol ligand was synthesized methods from the literature.[8]

Supplementary Note 2: Quantum dot core synthesis

Wurtzite (W) CdSe core synthesis

W CdSe QDs were synthesized using methods of Manna et al.[9] with minor modifications. In a typical synthesis, a Cd precursor solution was prepared by mixing CdO (60 mg), ODPA (280 mg), and TOPO (3 g) in a 50-mL round bottom flask (r.b.f.), dried under vacuum at ~100°C for 1 h, and heated to ~320°C under nitrogen until the brown mixture became a clear colorless solution. TOP (1 mL) was then added to the Cd solution and the temperature was stabilized at a desired value for the Se precursor injection (300–365°C). The Se precursor solution was made by sonicating a dispersion of Se powder (60 mg) in TOP (0.5 mL) until it became a clear solution. This Se/TOP solution was added with a controlled amount of 0.8 M DPP in TOP stock solution (10–100 µL). QDs were produced by injecting the Se solution into the Cd solution, and sizes were tuned by varying the injection temperature, amount of DPP/TOP stock solution, and the growth time. Detailed synthetic conditions are provided in Table S1. The QDs were purified by diluting the reaction mixture with toluene (3 mL) followed by precipitation with excess methanol (~40 mL). After two more cycles of dissolution in toluene and precipitation with methanol, purified QDs were dissolved in hexane and stored as a pure stock solution.

Zinc-blende (ZB) CdSe core synthesis

Diphenylphosphine selenide (DPPSe) synthesis. DPPSe was synthesized by reacting DPP with Se powder in 1:1 molar ratio under nitrogen at room temperature.

2.3 nm CdSe – CdO (0.6 mmol), TDPA (1.33 mmol), and ODE (27.6 mL) were mixed in a 250-mL r.b.f. and heated to ~320°C under nitrogen until the mixture became a clear colorless solution. HDA (7.1 g) was added and the temperature was stabilized at 300°C. A Se precursor solution was prepared by mixing a Se/TOP stock (1 M, 3 mL), DPPSe (52.5 mg), and TOP (4.5 mL) under nitrogen. QDs were grown by swiftly injecting the Se solution into the Cd solution with vigorous stirring using a 10-mL syringe with a wide bore (16 G) needle. 30s after the Se injection, the heating mantle was quickly removed and the reaction solution was rapidly cooled under a stream of air. The reaction solution was divided into two 50-mL tubes and QDs were precipitated by adding a mixture of methanol (15 mL) and acetone (15 mL). QDs were then redispersed in hexane and purified by methanol extraction. Finally, purified QDs dispersed in hexane were stored as a concentrated stock solution.

3.0 nm & 4.2 nm CdSe – CdSe QDs were synthesized using the method of Chen et al.[2] with some modifications. $Cd(BAc)_2$ (for 3.0 nm CdSe; 0.2 mmol) or Cd(MAc)2 (for 4.2 nm CdSe; 0.2 mmol), SeO_2 (0.2 mmol), HDD (0.2 mmol), and ODE (4 mL) were mixed in a 50-mL r.b.f. and dried under vacuum at ~100°C for 2 hours. Then the temperature was raised to 230°C at a rate of ~20°C/min under nitrogen. The solution color changed from colorless to pale yellow at ~190°C indicating CdSe nucleation. After reaching 230°C, the temperature was maintained for 15 min. QD growth was quenched by removing the heating mantle. When cooled to ~110°C, the reaction solution was mixed with chloroform (10 mL) containing OAc (1 mL) and OLA (0.6 mL). Purification was performed by precipitating the QDs through the addition of a mixture of methanol (15 mL) and acetone (15 mL). QDs were redispersed in hexane (~20 mL) and extracted twice with methanol (5–10 mL per cycle) followed by precipitation with excess methanol. Finally, QDs were washed with a few mL of acetone to ensure that there was no methanol remaining, and dispersed in hexane as a stock solution.

$CdSe_{0.42}S_{0.58}$ and $CdSe_{0.89}S_{0.11}$ alloy core synthesis

CdSeS alloy QDs were synthesized and purified by following the same protocol used for 3.0 nm CdSe QDs with a difference of using controlled ratios of the two chalcogen precursors, SeO_2 and elemental S, instead of using SeO_2 alone. For $CdSe_{0.42}S_{0.58}$ core synthesis, $Cd(BAc)_2$ (0.2 mmol), SeO_2 (0.066 mmol), S (0.134 mmol), and HDD (0.2 mmol) were reacted in ODE (4 mL) (Se : S = 0.33 : 0.67). For $CdSe_{0.89}S_{0.11}$ core (HgCdSeS-1 in the Supplementary Figure 10 & 11) synthesis, $Cd(BAc)_2$ (0.6 mmol), SeO_2 (0.4 mmol), S (0.2 mmol), and HDD (0.6 mmol) were reacted in ODE (12 mL) (Se : S = 0.67 : 0.33). Elemental analysis using an inductively coupled plasma–optical emission spectrometry (ICP-OES) system was used to confirm the Se-to-S ratios.

CdS core synthesis

CdS QDs were synthesized by adjusting the methods of Cao and coworkers.[2,3]

2.0 nm CdS – $Cd(BAc)_2$ (0.2 mmol), S (0.2 mmol), HDD (0.2 mmol), and ODE (4 mL) were mixed in a 50-mL r.b.f. and dried under vacuum at ~100°C for 2 hours. Then CdS QDs were grown by raising the temperature to 230°C at a rate of ~20°C/min under nitrogen. The temperature was maintained at 230°C for 15 min before cooled to ~110°C for purification. The purification procedure was the same as for the 3.0 nm CdSe synthesis.

2.8 nm CdS – TBPS was synthesized by sonicating S powder in TBP under nitrogen with 1:1 S-to-TBP molar ratio. $Cd(BAc)_2$ (0.2 mmol), HDD (0.2 mmol), and ODE (3.2 mL) were mixed in a 50-mL r.b.f. and dried under vacuum at ~100°C for 2 hours. TBPS (1.25 M) in an ODE stock solution (0.8 mL) was injected under nitrogen and the temperature was increased to 230°C at a rate of ~20°C/min. CdS nucleated at ~110°C. The temperature was maintained at 230°C for 30 min before cooled down to ~110°C for purification. The purification procedure was the same as for the 3.0 nm CdSe synthesis.

3.7 nm CdS – A solution of S in ODE was prepared by mixing S powder (1 mmol) with ODE (10 mL) under nitrogen and heating to ~200°C until the mixture became a clear colorless solution. $Cd(BAc)_2$ (0.2 mmol), S (0.2 mmol), 2,2'-dithiobisbenzothiazole (0.0625 mmol), HDD (0.2 mmol), and ODE (3.4 mL) were mixed in a 50-mL r.b.f. and dried under vacuum at ~100°C for 2 hours. Then CdS QDs were grown by heating the solution to 230°C at a rate of ~20°C/min under nitrogen. After keeping the temperature at 230°C for 15 min, the dropwise addition of 0.1 M S/ODE stock solution (1 mL) over ~40 min allowed additional particle growth.

HgCdSeS alloy core synthesis

HgCdSeS QDs were prepared through Hg cation exchange reactions on CdSe or CdSeS QDs using method developed by Smith & Nie[4,6] with several modifications.

Hg exchange using $Hg(OT)_2$ – A CdSe or CdSeS QD stock in hexane (~100 nmol in a few mL of hexane) was injected into OLA (5 mL) under nitrogen and hexane was evaporated completely under vacuum at 40–50°C. Hg exchange was initiated by adding $Hg(OT)_2$ (2x excess of total Cd atoms) either as powder or as a solution in OLA (0.1 M). The reaction rate was adjusted by gradually increasing the temperature (40–150°C). Changes in the bandgap energy and the absorption extinction were carefully monitored by removing precise aliquot volumes (typically 200 µL, then diluted 10-fold with chloroform) every 3–5 min to measure the UV–vis–NIR absorption spectrum. Detailed reaction parameters are provided in Table S2. When a desired

amount of redshift was induced, reaction quenching and purification were performed by precipitating the QDs through the addition of a 1:1 mixture of methanol /acetone (~30 mL total). The QD precipitate was washed two times with methanol then finally dispersed in hexane to be used as a stock solution. The stock solution was left at room temperature for at least a day before use in core/shell QD synthesis because there was typically an additional 10–15 nm redshift in the absorption spectra over time due to internal diffusion of Hg ions.

Hg exchange using $Hg(Ac)_2$ – CdSe QDs (100–200 nmol) were dispersed in a 0.2 M solution of OLA in chloroform (4–5 mL). A 0.1 M $Hg(Ac)_2(OLA)_2$ stock solution was prepared by dissolving $Hg(Ac)_2$ (0.5 mmol) in a 0.2 M OLA solution in chloroform (5 mL). Hg exchange was initiated by swiftly injecting the $Hg(Ac)_2(OLA)_2$ solution (3x excess of total Cd atoms) into the CdSe QD solution under vigorous stirring. The extent of Hg exchange was carefully monitored by taking absorption spectra of aliquots (~100 µL, then diluted 10-fold with chloroform) every 3–5 min. After the desired amount of redshift, the reaction was quenched by adding OT (~100 µL) and precipitating the QDs with a 1:1 mixture of methanol/acetone (~20 mL total). QDs were further purified by three cycles of redispersion in hexane (~10 mL) with OLA (~200 µL) and OAc (~100 µL) and precipitation with methanol/acetone. Finally, the QDs were dispersed in hexane and stored as a stock solution for at least a day before use in core/shell QD synthesis.

Supplementary Note 3: CdS and ZnS shell growth

CdS and ZnS shell growth was performed using a layer-by-layer shell growth protocol developed by Bawendi and coworkers[10] with some modifications.

Cd precursor solution – A Cd precursor solution was prepared by mixing CdO (1 mmol), OAc (2.1 mmol), and ODE (3.9 mL) and heating to ~250°C under nitrogen until the brown mixture became a clear solution. After cooling to ~100°C, DA (2 mmol) was added. Then the solution was diluted 1:1 with TOP.

Zn precursor solution – A Zn precursor solution was prepared by dissolving $Zn(Ac)_2$ (1 mmol) in OLA (10 mL) under nitrogen.

S precursor solution – A S precursor solution was prepared by dissolving $(TMS)_2S$ (0.5 mmol) in TOP (5 mL) under nitrogen.

Layer-by-layer shell growth – To prevent homogeneous nucleation of shell materials, CdS shell was grown as increments of 0.8 ML instead of 1 ML. 1 ML thickness was set to ~0.3 nm which is the thickness of a single CdS layer along the $(100)_{ZB}$ direction. The amount of precursors needed was calculated based on the volume increase by the shell growth in a single monolayer and the total number of cores in the solution. In a typical reaction, a purified core stock in hexane (50–100 nmol) was injected into a mixed solvent of ODE (2 mL) and OLA (1 mL) in a 50-mL r.b.f. and hexane was completely evaporated under vacuum at 40–50°C. Next, the solution was heated under nitrogen to the temperature used for the first 0.8 ML shell growth (typically 120–130°C for 2–3 nm cores and HgCdSeS cores, and 150–170°C for >4 nm cores). An aliquot (200 µL) was withdrawn using a glass microsyringe and diluted 10-fold in chloroform to monitor the reaction. The S precursor for the first 0.8 ML shell was dropwise added within 3–5 min and allowed to react for 15–20 min. The same amount of Cd precursor was added in the same manner and allowed to react for another 15–20 min to complete the first cycle. Another aliquot (200 µL) was withdrawn and diluted 10-fold in chloroform to measure the absorption and emission spectra, fluorescence quantum yield, and extinction coefficient. The 0.8 ML shell growth cycle was repeated as desired. The reaction temperature was raised stepwise by ~10°C

between each cycle until reaching a maximum of ~190°C. Aliquots were withdrawn after each cycle to monitor the optical property changes during shell growth.

After growing a desired amount of CdS shell, the metal precursor was switched to Zn to grow the ZnS shell, which was grown in either 0.5 ML (in Figure 4) or 0.8 ML (in Supplementary Figures 7 and 9) steps. The reaction temperature for ZnS shell growth was 200-220°C. Aliquots were similarly withdrawn after each 1 ML (in 0.5 ML step growth) or 0.8 ML (in 0.8 ML step growth) of shell growth.

The reaction was quenched by reducing the temperature. For purification, the reaction solution was diluted 2–3 fold in chloroform in a centrifuge tube and the QDs were precipitated by adding acetone. QDs were redispersed in chloroform and centrifuged at 7,000 g for 10 min to remove any undissolved byproducts. Then this chloroform solution was used for optical analysis and phase transfer.

First monolayer of CdS shell growth on HgCdSe cores using TOP-free Cd & S precursor solution – Because of the strong binding affinity of TOP toward mercury ions, TOP could degrade bare HgCdSeS QDs by extracting Hg ions out of the structure. Such extraction was accelerated at elevated temperatures. Thus, the first monolayer of CdS shell needed to be grown in a TOP-free solution and at relatively low temperature. The first 0.8 ML portion of TOP-free S precursor was added dropwise starting at ~50°C while slowly raising the temperature up to 120–130°C in ~5 min. After allowing the S precursor to react for 15–20 min at 130°C, TOP-free Cd precursor for the first 0.8 ML shell was added dropwise at ~140°C and allowed to react for another 15–20 min. Once HgCdSeS QDs were passivated by a full monolayer of CdS shell, they were stable against TOP so that the regular TOP-containing precursors could be used for the further shell growth.

A TOP-free Cd precursor solution was prepared by mixing CdO (1 mmol), OAc (2.1 mmol), and ODE (8.9 mL) and heating to ~250°C under nitrogen until the brown mixture became a clear solution. The solution was cooled to ~100°C and DA (2 mmol) was added. The solution was then cooled to room temperature.

A TOP-free S precursor solution was prepared by dissolving $(TMS)_2S$ (0.5 mmol) in ODE (5 mL) under nitrogen.

Supplementary Note 4: Quantum dot phase transfer

QD phase transfer with amphiphilic polymers. A purified core/shell QD dispersion in chloroform (~1 nmol/mL, 2–10 mL) was transferred to a vial with a stir bar. In a separate vial, amphipol (~200 mg) was dissolved in chloroform (10 mL) at room temperature. While vigorously stirring the QD dispersion, a 2,000–2,500x molar excess of amphipol was added dropwise. The vial was sealed with a septum screw cap and placed in a vacuum desiccator with a puncture on the cap using a disposable needle (20–22 G). Chloroform was slowly evaporated overnight under house vacuum while vigorously stirring the solution. After completely removing chloroform, 10 mM sodium hydroxide solution in distilled water was added (2–3 mL/nmol of QD) and stirred for several hours until amphipol-coated QDs were fully dispersed. Finally, the solution was centrifuged at 7,000 g for 10 min to remove any QD aggregates and used in optical characterization including brightness measurements in solution and at the single molecule level. For intravital imaging experiment, these amphipol-coated QDs were purified using a size-exclusion column and dialysis. Typically, 30–40 nmol of amphipol-coated QDs in 1x phosphate buffered saline (PBS) was injected into a GE ÄKTAprime plus chromatography system using a Superose 6 column with PBS eluent at a flow rate of 0.5 mL/min. This separated amphipol-

coated QDs with most of the free amphipol micelles. Then, these QDs were further purified by dialysis for 36 h in PBS using a 50 kDa dialysis tube.

PEG conjugation on polymer-coated QDs. Amphipol-coated QDs show strong nonspecific binding in biological systems due to the negatively charged carboxylic acid groups covering the surface. Therefore, for the intravital multiphoton imaging experiments, amphipol-coated QDs were conjugated with amino-polyethylene glycol (amino-PEG). PEG coating was performed by following a protocol in the literature.[6] Typically, amphipol-coated QD solutions in 1x PBS (~1 nmol/mL, ~10 mL) were mixed with 40,000x molar excess of 750 Da amino-PEG in DMSO (~0.5 mL) at room temperature. Then, a 25,000x molar excess of freshly prepared solution of DMTMM in DMSO (0.5 M) was injected into the QD–amino-PEG solution and stirred at room temperature for 30 min. This DMTMM addition and reaction was repeated 4 more times to maximize the PEG conjugation on QD surface. The reaction was quenched by adding 1M Tris buffer (pH ~8.5), and QDs were purified by dialysis in PBS for 24 h. Finally, PEG-coated QDs in PBS were centrifuged at 7,000g for 10 min to remove any aggregates and filtered using a 200 μm pore-size syringe filter.

QD phase transfer with multidentate polymers. Purified core/shell QDs dispersed in hexane were phase transferred to NMF with the addition of TMAH (100 equivalent to the QD surface atoms). The resulted OH- capped QDs in NMF were then mixed with a multidentate polymer (5 equivalent of to the QD surface atoms). The mixture was stirred for 2 h at 50°C under N_2. To remove excess free ligands and organic solvent, the QDs dispersion was first diluted with 50 mM sodium borate buffer (pH 8.5) and re-concentrated using an Amicon Ultra centrifugal filter (50 kDa MWCO). This dilution-concentration cycle was performed 4 more times.

QD phase transfer with hydrophilic thiols. Purified QDs in $CHCl_3$ were mixed with an aqueous solution of the thiol ligand. The biphasic mixture was stirred at 50°C for 2 h under N_2. Phase transfer from organic phase to aqueous phase was indicated by disappearance of fluorescence in the $CHCl_3$ phase. To remove excess free ligands and organic solvent, the QDs dispersion was first diluted with 1x PBS (pH 7.4) and then re-concentrated using an Amicon Ultra centrifugal filter (50 kDa MWCO). This dilution-concentration cycle was performed 4 more times.

Supplementary Note 5: Instrumentation

UV–Vis–NIR absorption spectroscopy. Absorption spectra of quantum dot solutions were obtained using a Agilent Cary 5000 UV–Vis–NIR spectrometer. If the solution was highly concentrated (e.g. QD solutions for elemental analysis), an aliquot was diluted 10 or 20 fold so that their absorbance was in the dynamic range of the spectrometer (absorbance < 4) in the entire spectral range (typically 200–800 nm).

Fluorescence and photoluminescence excitation (PLE) spectroscopy. Fluorescence and PLE spectra of a QD dispersion were obtained using a Horiba NanoLog spectrofluorometer. Dispersions were diluted enough to eliminate self-quenching of fluorescence (typically, absorbance @ 490nm < 0.1). Signal acquisition conditions such as scan time, slit widths, and number of scans were adjusted so that the brightest sample was not saturating the detector (photomultiplier tube) and all spectra showed sufficiently high signal-to-noise ratios. Raw fluorescence signal measured by the detector was corrected by both the wavelength-dependent detector sensitivity factor provided by the manufacturer and the excitation power fluctuation monitored by a built-in photodiode before they were used in fluorescence quantum yield and brightness calculations. PLE spectra were usually obtained by fixing the detection wavelength at

the fluorescence peak maximum scanning the excitation wavelength from ~300 nm up to 10–30 nm less than the detection wavelength.

Transmission electron microscopy (TEM). TEM images of QDs were obtained using a JEOL 2010 LaB_6 high-resolution microscope in the Frederick Seitz Materials Research Laboratory Central Research Facilities at the University of Illinois. Samples were prepared by placing a drop of dilute QD solution in hexane or chloroform on an ultrathin carbon film TEM grid (Ted Pella, Product# 01824) and then wicking the solution off with a tissue.

Inductively coupled plasma–optical emission spectrometry (ICP–OES). Elemental analysis was performed with a PerkinElmer Optima 2000DV ICP–optical emission spectrometer in the Microanalysis Laboratory at the University of Illinois. Samples were prepared by digesting QDs with nitric acid under high pressure (60 bar) in a PerkinElmer/Anton Parr Multiwave 3000 microwave digester. Typically, a concentrated well-purified QD solution in hexane (band edge absorption near 30–40) was prepared and its absorption spectrum was carefully measured to calculate the extinction coefficient. Then, the solution (1 mL) was transferred to a Teflon tube and hexane was completely evaporated under nitrogen flow. Three identical samples were prepared simultaneously for a precise measurement. QDs were digested into ions in the microwave reactor and the entire product was diluted to exactly 20 mL in distilled water before injection into the ICP–OES spectrometer.

Fluorescence microscopy. All samples were imaged via wide-field illumination on a Zeiss Axio Observer.Z1 inverted microscope with a 100x 1.45 NA alpha Plan-Fluar oil immersion microscope objective with 100 W halogen lamp illumination. Excitation light was filtered using a 390/40 bandpass filter (Semrock Inc.), and emission light was filtered with a 496 longpass filter (Semrock Inc.). Images were acquired using a Photometrics eXcelon Evolve 512 EMCCD through Zeiss Zen software. All samples were uniformly excited and data was collected for 30 seconds at a rate of 19.4 frames/s. Excitation power was acquired using a PM121 optical power meter (Thor Labs).

Multiphoton fluorescence brightness measurement. All samples were measured using a Zeiss 710 confocal scanner Azio Observer.Z1 inverted microscope with a 10x 0.30 NA EC Plan-Neofluar microscope objective with tunable Mai-Tai Ti-Sapphire laser (Spectra Physics) excitation. Laser power was acquired using a PM121 optical power meter. Spectrally resolved emission spectra were acquired using a Zeiss 34-Channel QUASAR detection unit.

Supplementary Note 6: Extinction coefficient measurements

Calculation of extinction coefficient (ε) and absorption coefficient (α)

Extinction coefficients, ε ($cm^{-1}M^{-1}$), of QDs were calculated using the Beer–Lambert law of absorbance described in eq. 1,

$$\varepsilon = \frac{A}{l \cdot c_{\mathrm{QD}}} \tag{1}$$

where A is the absorbance of a QD solution (unitless), l is the path length (cm), and c_{QD} is the concentration of QD (M). A of a QD solution was directly measured using UV-vis-NIR absorption spectrophotometry. l was determined by the dimension of the cuvette holding the solution in the beam path. c_{QD} was derived from two independent measurements: average QD size (radius), r (nm), obtained by transmission electron microscopy (TEM) and elemental concentration of Cd in the solution, c_{Cd} (M), acquired from elemental analysis. Then, r is used to calculate the average

number of Cd atoms in a single QD, n_{Cd}, relying on the assumption that all QDs are spherical and have density of the bulk material, d_{Bulk}, as expressed in eq. 2,

$$n_{\mathrm{Cd}} = \frac{\frac{4\pi}{3} r^3 \cdot d_{\mathrm{Bulk}} \cdot N_{\mathrm{A}}}{M} \tag{2}$$

where, M is the molecular weight of the material (g·mol^{-1}) and N_{A} is the Avogadro constant (6.022 × 10^{23} mol^{-1}). Then c_{Cd} can be converted to c_{QD} by eq. 3,

$$c_{\mathrm{QD}} = \frac{c_{\mathrm{Cd}}}{n_{\mathrm{Cd}}} \tag{3}$$

The absorption coefficient, α (cm^{-1}), was then derived from the absorption extinction coefficient by the relationship given in eq. 4,[11]

$$\alpha = \frac{1000 \cdot \ln(10)}{\frac{4\pi}{3} r^3 N_{\mathrm{A}}} \varepsilon \tag{4}$$

ε and α of CdSe, CdS and CdSeS cores were obtained by carrying out the above steps. Whereas, those of HgCdSe(S) alloy cores and all core/shell QDs were acquired by carefully measuring the changes in absorption spectra during Hg cation exchange and shell growth reactions, respectively, based on the assumption that total QD concentration remains constant through the reaction.

Supplementary Note 7: Quantum yield measurements

Detailed mathematical formulations and experimental protocols for fluorescence quantum yield (QY) measurements are well described in the literature.[11–13] The QYs of our QD samples were obtained by following standard relative QY measurement protocols described in those literature reports. This section briefly covers the basic equations necessary for QY calculation and discusses in detail the protocols for excitation energy-dependent QY measurement of QD samples.

Relative QY calculation

QY of a fluorophore (Φ_{f}) is defined as the ratio of the number of emitted photons (N_{Em}) to the number of absorbed photons (N_{Abs}) as eq. 5,[12]

$$\Phi_{\mathrm{f}} = \frac{N_{\mathrm{Em}}}{N_{\mathrm{Abs}}} \tag{5}$$

QY of a fluorescent sample is often determined by comparing its fluorescence with that of a reference with known QY (e.g. molecular dyes) both measured using the same instrumental setup. The ratio between the QY of a sample excited at $\lambda_{\mathrm{Ex,x}}$ ($\Phi_{\mathrm{f,x}}(\lambda_{\mathrm{Ex,x}})$) and that of a reference excited at $\lambda_{\mathrm{Ex,Ref}}$ ($\Phi_{\mathrm{f,Ref}}(\lambda_{\mathrm{Ex,Ref}})$) can be given using eq. 6,

$$\frac{\Phi_{\mathrm{f,x}}(\lambda_{\mathrm{Ex,x}})}{\Phi_{\mathrm{f,Ref}}(\lambda_{\mathrm{Ex,Ref}})} = \frac{\dfrac{F_{\mathrm{x}}(\lambda_{\mathrm{Ex,x}})}{q_{\mathrm{p}}(\lambda_{\mathrm{Ex,x}}) f_{\mathrm{x}}(\lambda_{\mathrm{Ex,x}})}}{\dfrac{F_{\mathrm{Ref}}(\lambda_{\mathrm{Ex,Ref}})}{q_{\mathrm{p}}(\lambda_{\mathrm{Ex,Ref}}) f_{\mathrm{Ref}}(\lambda_{\mathrm{Ex,Ref}})}} \frac{n_{\mathrm{x}}^2}{n_{\mathrm{Ref}}^2} \tag{6}$$

where the subscript "x" and "Ref" denote the sample and reference, respectively, $F(\lambda_{\text{Ex}})$ is the *integrated fluorescence photon flux* with excitation at λ_{Ex}, $q_{\text{P}}(\lambda_{\text{Ex}})$ is the *excitation photon flux* at λ_{Ex}, $f(\lambda_{\text{Ex}})$ is the *absorption factor* at λ_{Ex}, and n is the *refractive index* of the solvent. Therefore eq. 6 indicates that N_{Em} and N_{Abs} are proportional to $F(\lambda_{\text{Ex}})$ (total amount of emitted photon flux) and $q_{\text{P}}(\lambda_{\text{Ex}}) \times f(\lambda_{\text{Ex}})$ (total amount of excited photons), respectively, and the refractive index difference needs to be considered when comparing two different fluorophores.

$F(\lambda_{\text{Ex}})$ is the fluorescence photon flux generated by exciting the fluorophore at λ_{Ex} and measured at λ_{Em} ($q_{\text{P},\lambda\text{Ex}}^{\text{f}}(\lambda_{\text{Em}})$) integrated over the entire emission spectrum (λ_{a} □ λ_{Em}□ λ_{b}) as in eq. 7,

$$F(\lambda_{\text{Ex}}) = \int_{\lambda_a}^{\lambda_b} q_{\text{p},\lambda_{\text{Ex}}}^{\text{f}}(\lambda_{\text{Em}})d\lambda_{\text{Em}} = \int_{\lambda_a}^{\lambda_b} \frac{I_{\lambda_{\text{Ex}}}(\lambda_{\text{Em}})}{s(\lambda_{\text{Em}})} \frac{\lambda_{\text{Em}}}{hc} d\lambda_{\text{Em}} \tag{7}$$

$q_{\text{P},\lambda\text{Ex}}^{\text{f}}(\lambda_{\text{Em}})$ is the emission intensity measured at λ_{Em} ($I_{\lambda\text{Ex}}(\lambda_{\text{Em}})$) corrected by the wavelength-dependent responsivity of the detector ($s(\lambda_{\text{Em}})$). Since the QY is the ratio between the number of photons, $I(\lambda_{\text{Em}})$ should be presented as a photonic quantity (e.g., photon counts per second (cps)). If $I(\lambda_{\text{Em}})$ is measured as a radiometric quantity (e.g. W/s), it should be converted to a photonic quantity by dividing with $\frac{hc}{\lambda_{\text{Em}}}$ (h: Plank constant; c: speed of light), the energy of a photon with wavelength λ_{Em}.

$f(\lambda_{\text{Ex}})$ is defined as the fraction of excitation photons absorbed by the sample at λ_{Ex} which can be formulated in terms of transmittance ($T(\lambda_{\text{Ex}})$) or absorbance ($A(\lambda_{\text{Ex}})$) using eq. 8,

$$f(\lambda_{\text{Ex}}) = 1 - T(\lambda_{\text{Ex}}) = 1 - 10^{-A(\lambda_{\text{Ex}})} \tag{8}$$

$q_{\text{P}}(\lambda_{\text{Ex}})$ is excitation source intensity at λ_{Ex} measured by photodetector are corrected by the wavelength-dependent sensitivity of the detector as in the emission photon flux calculation. Also it must be read as or converted to a proper photonic quantity that is linearly proportional to the number of excitation photons.

Excitation wavelength-dependent quantum yield calculation using the photoluminescence excitation spectrum

Photoluminescence excitation (PLE) spectra provide the change in the fluorescence intensity at a specific emission wavelength ($\lambda_{\text{Em Max}}$) depending on the excitation wavelength, or a plot of $q_{\text{P},\lambda\text{Ex}}^{\text{f}}(\lambda_{\text{Em}}^{*})$ versus λ_{Ex}. If the shapes (e.g. λ_{EmMax}, FWHM) of the fluorescence spectra obtained at different excitation wavelengths are identical, the integrated fluorescence photon flux $F(\lambda_{\text{Ex}})$ can simply be derived from the PLE spectrum and one $F(\lambda_{\text{Ex}})$ measured at a reference excitation wavelength according to eq. 9,

$$F(\lambda_{\text{Ex}}) = \frac{q_{\text{p},\lambda_{\text{Ex}}}^{\text{f}}(\lambda_{\text{Em}}^{*})}{q_{\text{p},\lambda_{\text{Ex Ref}}}^{\text{f}}(\lambda_{\text{Em}}^{*})} F(\lambda_{\text{Ex Ref}}) \tag{9}$$

Absorption factor (f) vs absorbance (A) in quantum yield calculation

For very dilute samples ($A < 0.1$), the absorption factor $f(\lambda_{\text{Ex}})$ is often replaced by absorbance $A(\lambda_{\text{Ex}})$ by using a power series expansion as shown in eq. 9 and eq. 10.

$$10^{-A} = \sum_{n=0}^{\infty} \frac{1}{n!} \frac{d^n}{dA^n} (10^{-A}) = \sum_{n=0}^{\infty} \frac{(-\ln 10)^n}{n!} A^n \qquad (10)$$

$$f(\lambda_{\mathrm{Ex}}) = 1 - 10^{-A(\lambda_{\mathrm{Ex}})} = 1 - (1 - 2.3026 A(\lambda_{\mathrm{Ex}}) + \frac{2.3026^2}{2} A^2(\lambda_{\mathrm{Ex}}) - ...) \approx 2.3026 A(\lambda_{\mathrm{Ex}}) \qquad (11)$$

The constant 2.3026 is dropped off when calculating the ratio between absorbance of a reference and a sample for QY calculation. However, this approximation is accurate only when the absorbance values of both the reference (A_{Ref}) and sample (A_{x}) are very low and *close to each other*. Panel a of the figure below shows the difference between the QY calculated by using the ratio of $A_{\mathrm{x}}/A_{\mathrm{Ref}}$ and that calculated by using $f_{\mathrm{x}}/f_{\mathrm{Ref}}$. Notice that there can be up to 10% error in QY from absorbances even when both A_{Ref} and A_{x} are lower than 0.1 but the values are different (e.g. A_{Ref} = 0.01 and A_{x} = 0.1). Moreover, such deviation quickly becomes enormous when the absorbance of a sample further increases relative to the absorbance of the reference. In fact, this is generally the case for calculating an excitation wavelength-dependent QY of a QD sample from its PLE spectrum. Although for a dilute QD solution with an absorbance < 0.1 near the bandedge, the solution can still show very high absorbance as the wavelengths gets shorter due to the band-type electronic structure of a QD as shown in panel b. Hence, it is unavoidable that the sample is excited at regions where the sample absorbance is much higher than the reference absorbance when collecting a PLE spectrum, and there can be a significant error when absorbance is used instead of absorption factor in the QY calculation.

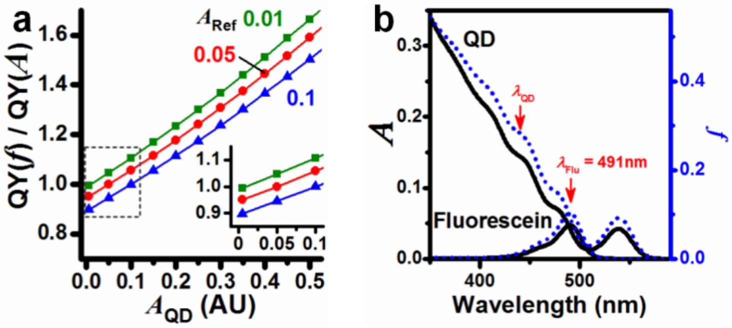

Measurement of excitation wavelength-dependent quantum yield of quantum dots

Sample preparation: A fluorescein solution in 1 mM NaOH water ($\Phi_{\mathrm{f,Ref}}$ = 0.92; n = 1.333) was used as the QY reference.[13-14] The fluorescein absorbance at the lowest energy absorption peak (490 nm) was adjusted to 0.03–0.05. QD sample solutions were prepared in either chloroform (organic soluble QDs; n = 1.445) or 10 mM NaOH water (amphipol-coated water-soluble QDs; n = 1.333). QD solutions were centrifuged to remove any QD aggregates or undissolved debris that may induce scattering. Then the solutions were diluted to make the absorbance at 490 nm 0.03–0.05.

Relative quantum yield measurement: The absorption spectrum of a sample or the reference was first obtained by absorption spectrophotometry. The spectrum was then converted to an absorption factor spectrum for the QY calculation. The emission spectrum was obtained by exciting the sample either at 490 nm (λ_{Ex} of fluorescein) or 400 nm. Data acquisition conditions such as excitation & emission slit width, emission acquisition time, and number of scans were adjusted to obtain the signals with the highest signal-to-noise ratio within the dynamic range of the detectors. Then the condition was kept the same for all samples and the reference. The emission intensity was recorded in cps unit (photonic scale). The spectrum was corrected by the blank spectrum of solvent then multiplied by the wavelength-dependent sensitivity correction

factor for the detector acquired from the manufacturer to represent the emission photon flux. Then, this corrected emission spectrum was integrated over the entire emission wavelength range to calculate the total fluorescence photon flux ([illegible] N_{Em}). The photon flux of the excitation source was monitored simultaneously by a silicon photodiode built in the sample compartment of the fluorometer. The diode read the relative photon flux in microAmp unit (a photonic scale) and it was also corrected by its wavelength-dependent sensitivity given by the manufacturer. Then, excitation photon flux was multiplied by the absorption factor at the same wavelength ([illegible] N_{Abs}), and used to normalize the total fluorescence photon flux ([illegible] $N_{\mathrm{Em}}/N_{\mathrm{Em}}$). Finally, QY was determined by calculating the ratio between this normalized quantity of a sample against that of the fluorescein reference. A PLE spectrum was obtained by monitoring the emission signal at the peak maximum and sweeping the excitation wavelength (typically from ~350 nm up to 20–40 nm shorter than the peak maximum. Both emission and excitation photon fluxes were corrected by the detector sensitivities and the PLE curve was obtained by plotting the emission photon flux divided by the excitation photon flux against the excitation wavelength. Excitation wavelength-dependent QY was then calculated by dividing the PLE spectra with the absorption factor spectra.

Supplementary Note 8: Single particle brightness measurement

QD Sample preparation. Amphipol-coated QDs dissolved in 10 mM sodium hydroxide buffer were transferred to 1X PBS solution at a concentration of 1 µM and allowed to incubate at room temperature for 30 minutes. Afterward, the QDs were centrifuged at 7000g for 10 minutes in order to remove any aggregated particles. For imaging, QDs were nonspecifically adhered to #1.5 glass coverslips by spin-coating femtomolar dilutions at 2500 rpm for 30 seconds. Prior to spin-coating, the coverslips were rinsed with ethanol, methanol, and acetone in order to remove any organic residue.

Image analysis. Epifluorescence videos of single particles were saved as TIFF stacks and imported into Matlab for analysis using custom codes based on previous reports on single fluorophore analysis.[15-17] First, (*i,j*) coordinates of fluorescent spots were obtained from integrated images of all frames of each stack by calling the detection/estimation/deflation algorithm of Serge et al.[16] Using these positions, the fluorescence intensities of the detected QDs for each frame were measured by slight modifications to the methods of Arnspang et al.,[17] using the average value of a 3x3 pixel array centered on the detected position. Histograms of intensity value per frame (frequency vs. intensity) were then constructed for each detected QD, and the histograms were fit to a sum of two functions, a Gaussian background ($f_1(x)$) and an asymmetric Gaussian signal ($f_2(x)$).

$$f(x) = f_1(x) + f_2(x) \tag{12}$$

$$f_1(x) = \frac{\alpha_1}{\sigma_1\sqrt{2\pi}} \, exp\left[-\frac{1}{2}\left(\frac{x - x_1}{\sigma_1}\right)^2\right] \tag{13}$$

$$f_2(x) = \frac{1}{r+1}\frac{\alpha_2}{\sigma_2\sqrt{2\pi}} \, erfc\left(-\frac{x - x_1}{\sigma_1\sqrt{\pi}}\right) \square \begin{cases} exp\left[-\frac{1}{2r}\left(\frac{x - x_2}{\sigma_2}\right)^2\right], & for\ x < x_2 \\ exp\left[-\frac{1}{2}\left(\frac{x - x_2}{\sigma_2}\right)^2\right], & for\ x \geq x_2 \end{cases} \tag{14}$$

Here α, x, and σ are the integrated area, the centroid position in intensity, and the width of the Gaussian function, respectively. The subscript "1" corresponds to the QD state when it is entirely "off" (the noise level), and the subscript "2" corresponds to the QD state when it is "on." The asymmetry factor r, for which $0 < r \leq 1$, signifies that a single isolated QD has a maximum intensity value of fluorescence which can be fit as a Gaussian, however random blinking events yield lower intensity intermediate states that skew the distribution toward the low-intensity side of the function.

The function was fit to the data using the least squares method, yielding the following parameters for each detected particle: α_1, x_1, σ_1, α_2, x_2, σ_2, and r, as well as relative variances for each parameter (e.g. $RV(\alpha_1)$). The important parameters to extract, as displayed in Figure 5 of the main text are x_1 and x_2, the noise and signal intensities per particle, plotted as a histogram across the QD population. At this point of analysis, all spots had been detection without preference, and as such many comprised particles with overlapping point spread functions. To maximize the likelihood of only detecting individual QDs, we imposed the following criteria to the data obtained for all particles detected, chosen to select particles exhibiting conventional single-molecule behavior. (1) QDs "on" for at least 20% of frames, or $\alpha_2 \ / \ (\alpha_1 + \alpha_2) \geq 0.20$, (2) "on" for no more than 92% of frames, or $\alpha_2 \ / \ (\alpha_1 + \alpha_2) \leq 0.92$ so that a distinct

“off” level could be determined, (3) noise signal width less than 3.6, or $\sigma_1 < 3.6$, to avoid poor noise level fits, which were common for large particle aggregates (average σ in non-QD regions was 1.46 ± 0.40 in our measurements), (4) relative variance for all fitting parameters less than 300%. Typically 40% of detected QD points were rejected based on these criteria, and importantly, all of these criteria were based on goodness of fit to the mathematical model and confidence of detecting the correct fit parameters, and not based on the absolute intensity values, so that our detected brightness values were not skewed. Histograms of the values of x_1 and x_2 are depicted in Figure 5, after correcting the signal levels by the wavelength-specific sensitivity of the CCD camera.

Supplementary Note 9: Intravital microscopy

Animals. All procedures were conducted in accordance with the National Institutes of Health regulations and approved by the Albert Einstein College of Medicine animal use committee. PyMT tumor tissue from MMTV-PyMT (FVB mice) was cut into pieces of 2 to 3 mm and coated in matrigel (BD Biosciences, Franklin Lakes, NJ, USA). One piece of tumor was surgically implanted in the right lower mammary fat pads of FVB mice. After 4 – 6 weeks, when tumors are approximately 1 cm in diameter, live images of the tumor microenvironment were obtained using the skin flap procedure.[18]

***In vivo* multiphoton microscopy**. Intravital imaging of PyMT tumor-bearing mice was performed using methods similar to those previously described.[18] Images were acquired on a custom-built multiphoton microscope Olympus IX-71 with a 20X 0.95 NA water immersion objective and a tunable femtosecond laser (Mai Tai, Newport/Spectra-Physics)[19] tuned to 780 nm for optimal excitation of QDs. The fluorescence and second-harmonic signals generated were collected via a dichroic mirror and sent to three photomultiplier-tube (PMT) detectors to allow detection of second harmonic generation (SHG), CFP (from tumor cells) and QDs in green and red. Images were acquired from 3 random 512 x 512 pixels at a depth of 50 µm (21 slices at steps of 2 µm).

Image analysis. As previously described, image channels were balanced and subtracted to isolate the CFP signal.[18] An average intensity Z-projection was made for all channels. The average intensity projection is used for QD fluorescence to quantitatively determine the mean within a volume of interest. Five ROI were measured for each animal. The average pixel intensity of each ROI in the green channel was normalized to the average pixel intensity of the same ROI in the red channel. A single optical plane is presented in Figure 6 in the main text.

Supplementary References

1. Hoy, J. *et al*. Excitation energy dependence of the photoluminescence quantum yields of core and core/shell quantum dots. *Phys. Chem. Lett.* **4**, 2053-2060 (2013).
2. Chen, O. *et al*. Synthesis of metal-selenide nanocrystals using selenium dioxide as the selenium precursor. *Angew. Chem. Int. Ed.* **43,** 8638–8641 (2008).
3. Cao, Y. C. & Wang, J. One-pot synthesis of high-quality zinc-blende CdS nanocrystals. *J. Am. Chem. Soc.* **126,** 14336–14337 (2004).
4. Smith, A. M. & Nie, S. Bright and compact alloyed quantum dots with broadly tunable near-infrared absorption and fluorescence spectra through mercury cation exchange. *J. Am. Chem. Soc.* **133,** 24–26 (2011).
5. Gohon, Y. *et al.* Partial specific volume and solvent interactions of amphipol A8-35. *Anal. Biochem.* **334,** 318–334 (2004).
6. Carbone, L. *et al*. Synthesis and micrometer-scale assembly of colloidal CdSe/CdS nanorods prepared by a seeded growth approach. *Nano Lett.* **7,** 2941–2950 (2007).
7. Smith, A. M. & Nie, S. Compact quantum dots for single-molecule imaging in live cells. *J. Vis. Exp.* e4236 (2012).
8. Liu, W. *et al.* Compact biocompatible quantum dots via RAFT-mediated synthesis of imidazole-based random copolymer ligand. *J. Am. Chem. Soc.* **132,** 472–483 (2010).
9. Park, J. *et al*. Compact and Stable Quantum Dots with Positive, Negative, or Zwitterionic Surface: Specific Cell Interactions and Non-Specific Adsorptions by the Surface Charges. *Adv. Funct. Mater.* **21,** 1558–1566 (2011).
10. Greytak, A. B. *et al*. Alternating layer addition approach to CdSe/CdS core/shell quantum dots with near-unity quantum yield and high on-time fractions. *Chem. Sci.* **3,** 2028–2034 (2012).
11. Jasieniak, J., Smith, L., van Embden, J., Mulvaney, P. & Califano, M. Re-examination of the size-dependent absorption properties of CdSe quantum dots. *J. Phys. Chem. C* **113,** 19468–19474 (2009).
12. Demas, J. N. & Crosby, G. A. The measurement of photoluminescence quantum yields. A review. *J. Phys. Chem.* **75,** 991–1024 (1971).
13. Grabolle, M. *et al*. Determination of the fluorescence quantum yield of quantum dots: suitable procedures and achievable uncertainties. *Anal. Chem.* **81,** 6285–6294 (2009).
14. Wűrth, C., Grabolle, M., Pauli, J., Spieles, M. & Resch-Genger, U. Relative and absolute determination of fluorescence quantum yields of transparent samples. *Nat. Protoc.* **8,** 1535–1550 (2013).
15. Jaqaman, K. *et al*. Robust single-particle tracking in live-cell time-lapse sequences. *Nat. Methods.* **5**, 695-702 (2008).
16. Serge, A., Bertaux, N., Rigneault, H. & Marguet, D. Dynamic multiple-target tracing to probe spatiotemporal cartography of cell membranes. *Nat. Methods* **5**, 687-694 (2008).
17. Arnspang, E.C., Brewer, J.R. & Lagerholm, B.C. Multi-Color Single Particle Tracking with Quantum Dots. *PLoS ONE* **7**, e48521 (2012).
18. Wyckoff, J., Gligorijevic, B., Entenberg, D., Segall, J. & Condeelis, J. High-Resolution Multiphoton Imaging of Tumors In Vivo. *Cold Spring Harb. Protoc.* **2011**, 1167-1184 (2011).
19. Entenberg, D. *et al.* Setup and use of a two-laser multiphoton microscope for multichannel intravital fluorescence imaging. *Nat. Protoc.* **6**, 1500-1520 (2011).
